# Raising awareness and education of genetic testing and counseling through fotonovelas among Latina women at risk for hereditary breast and ovarian cancer

**DOI:** 10.1007/s12687-024-00728-5

**Published:** 2024-09-06

**Authors:** Rolando Barajas, Clara B. Barajas, Yaideliz M. Romero Ramos, Sara Gómez Trillos, Sabrina Sawhney, Claudia Campos, Alejandra Hurtado-de-Mendoza, Melissa Rotunno, Elizabeth Gillanders

**Affiliations:** 1 Georgetown University School of Medicine, Washington, DC USA; 2 Cancer Prevention and Control, Georgetown University, Lombardi Comprehensive Cancer Center, Washington, DC USA; 3 Department of Epidemiology, Brown University School of Public Health, Providence, RI USA; 4 Fischer Center for Hereditary Cancers, Washington, DC USA; 5 Nueva Vida, Inc, Alexandria, VA USA; 6 Division of Cancer Control and Population Sciences, Genomic Epidemiology Branch, National Health Institutes/National Cancer Institute, Bethesda, MD USA

**Keywords:** HBOC, Education, Hispanic women, Genetic services, GCRA

## Abstract

Latinas are less likely to receive genetic counseling and genetic testing (GCT) compared to non-Latina Whites because of systemic and patient-level barriers. We developed and tested fotonovelas to increase awareness of GCT among Latinas at-risk of hereditary breast and ovarian cancer (HBOC). Content for the fotonovelas was drawn from an existing culturally targeted narrative video focused on improving GCT use among Latinas at-risk of HBOC. Using mixed methods, we interviewed cancer patients (*n* = 10) and their relatives (*n* = 10) to assess the preliminary efficacy of the fotonovelas through pre-and post-fotonovela items assessing self-rated knowledge of GCT and willingness to discuss cancer with family. Health workers (*n* = 10) provided feedback on the fotonovela content. McNemar’s test was used to examine differences in the proportions of the outcomes pre- and post-fotonovelas. Interviews were transcribed and coded in Dedoose using a consensual qualitative research approach. Reading the fotonovelas increased self-rated knowledge of GCT by 22% (*p* = 0.16), from 50 to 60% in patients and from 63 to 100% among relatives. Analogously, reading the fotonovela increased willingness to talk about cancer with family by 33% (*p* = 0.02), from 70 to 100% in patients and from 38 to 75% in relatives. We identified six themes, some centered around the fotonovela’s message, feedback, and perceived barriers to GCT. Overall, participants liked the use of fotonovelas to increase GCT awareness and cancer conversations with family. Fotonovelas could potentially be used as educational tools to increase GCT awareness and cancer conversations among Latino families at-risk of HBOC.

**Supplementary Information:**

The online version contains supplementary material available at 10.1007/s12687-024-00728-5.

## Introduction

Latinas in the United States are more likely than non-Latina Whites to be diagnosed with breast cancer at advanced stages, with breast cancer subtypes that are more aggressive (e.g., triple-negative, and HER2-overexpressing tumors) (Zavala et al. 2021; Giaquinto et al. 2022; Swami et al. 2022). The most common cause of hereditary breast and ovarian cancer (HBOC) is the pathogenic variants in the *BRCA1* and *BRCA2* (*BRCA1/2*) genes. The lifetime risk of breast cancer ranges from 55 to 65% for *BRCA1* carriers and from 45 to 47% for *BRCA2* carriers (PDQ Cancer Genetics Editorial Board 2023). Similarly, the lifetime risk of ovarian cancer is 39% for *BRCA1* carriers and ranges from 11 to 17% for *BRCA2* carriers (PDQ Cancer Genetics Editorial Board 2023). Genetic cancer risk assessment (GCRA) is recommended for women with a personal or family history of breast or ovarian cancer who are at risk of carrying deleterious mutations of the *BRCA1/2* genes (Daly et al. 2021; US Preventive Services Task Force et al. 2019). GRCA includes genetic counseling and consideration of genetic testing if appropriate based on the patient’s history of hereditary cancer. However, Latina women have a lower level of awareness about genetic counseling and testing for inherited cancer risk (Wideroff et al. 2003; Mai et al. 2014; Hann et al. 2017; Hamilton et al. 2016) and lower use of GCRA compared to other populations (Hong et al. 2023).

Previous studies have found that Latina women experience several barriers to accessing genetic services. Some of these barriers include high cost and lack of health insurance (Hurtado-de-Mendoza et al. 2018), medical mistrust (Hann et al. 2017), unfamiliarity with genetic tests (Gómez-Trillos et al. 2019), and limited availability of bilingual genetic professionals (Augusto et al. 2019). Yet, when the process and advantages of GCRA are explained to Latina women, they show interest in the genetic services (Ricker et al. 2006; Komenaka et al. 2016; Gómez-Trillos et al. 2019; Dron et al. 2023). Therefore, ensuring that GCRA information is culturally tailored for Latina women at-risk for HBOC is critical to overcoming some of the barriers to genetic counseling and testing. Additionally, studies have documented that some Latina women at risk for HBOC prefer educational materials to be in Spanish, include concise information, and have a narrative or story format (Joseph et al. 2017; Dron et al. 2023; Hurtado-de-Mendoza et al. 2020).

Storytelling and narratives provide a way of remembering information through an individual’s connection to a story (Larkey and Gonzalez 2007; Kreuter et al. 2007). We sought to develop a fotonovela to raise awareness about genetic counseling and testing (GCT) among Latinas at risk for HBOC. Fotonovelas differ from common educational materials in format (e.g., formatted as a comic book) and present a short narrative with engaging visual elements and simple text (Valle, Yamada, and Matiella 2006; Cabassa, Molina, and Baron 2012). The use of fotonovelas as a health-related educational tool to reach the Latino population has been increasing (Gallagher-Thompson et al. 2015; Hinojosa et al. 2011; Levitz et al. 2023; Nelson et al. 2008; Sanchez et al. 2021). A recent study also recommended the use of narrative messages to make genetic information more accessible (Hovick et al. 2022). However, to our knowledge, no fotonovelas geared toward Spanish-speaking Latina women have been used to raise awareness of GCRA services. In this paper, we describe the development of fotonovelas and preliminary results on the efficacy of the fotonovelas in raising awareness of GCT.

## Methods

### Study collaborators

This study was informed by the research team and two partners from a community-based organization (hereinafter referred to as the study team). The work of our academic and community partners focused on cancer prevention and control, which made them valuable collaborators in the study. The study was deemed exempt from review by the Institutional Review Board at the National Cancer Institute.

### Development of the fotonovelas

Fotonovelas were created to raise awareness of GCT among Latina women at risk for HBOC. The content of the fotonovelas was drawn from an existing culturally targeted narrative video, *“Is my Cancer Hereditary? Rosa visits a Genetic Counselor”* (Hurtado-de-Mendoza et al. 2019, 2020). This 18-minute video shows a story about Rosa, a Latina and breast cancer survivor diagnosed under 50 years of age. Rosa learns about her risk of HBOC and overcomes barriers to attending genetic counseling. The video shows four main scenes, one in which Rosa learns about HBOC from a doctor, a genetic counseling visit, another in which she shares the information learned with her family, and another with her friend. Further details on the theoretical framework and formative work that guided the video development are presented elsewhere (Hurtado-de-Mendoza et al. 2020). Of note, the term “genetic counselor” was translated into Spanish for the film as “consejera genetica,” and this term was used throughout the fotonovelas. However, the term “asesora genetica” is commonly used throughout Latin America.

Multiple iterations of screenshots from the video were selected (varying angles, close-ups, faces, dialogue, etc.) for the fotonovelas until three final scenarios were agreed upon by the study team as a base to create three distinct fotonovelas. Following the primary scenes of the video, the first fotonovela emphasizes the doctor-patient relationship, where Rosa goes to a doctor’s office and learns about GCT (referred to as the doctor fotonovela). In the second fotonovela, Rosa has dinner with her husband, children, and sister, and shares what she learned about genetic counseling (family fotonovela). The third fotonovela depicts a friend-to-friend conversation about cancer, where Rosa tells a friend about GCT (friend fotonovela). The study team felt that these three scenarios represented the most natural conversations to occur for a newly diagnosed patient. In addition, the conversation with the genetic counselor would have required a longer explanation because the counselor would have gone into detail about the patient’s family history of cancer before making a recommendation for testing.

With the overall goal of increasing knowledge about GCT, the fotonovelas had the same educational content. Specifically, the fotonovelas aimed to cover the following topics: hereditary cancer, genetic testing, the importance of knowing one’s family history of cancer, and understanding that a genetic counselor may recommend genetic testing based on a family history of cancer. In addition, the fotonovelas aimed to discuss common barriers to genetic counseling, such as concerns about cost and insurance coverage. The fotonovelas also aimed to explain the meaning of positive and negative genetic test results and to clarify that genetic testing is not a cancer diagnostic test. Also, that a Pap test is different from a genetic test. The fotonovelas were formatted in a comic book style, each ranging from 3 to 4 pages long (English and Spanish versions of the fotonovelas are available in Appendix 1).

### Participant eligibility criteria and recruitment

#### Cancer patients/survivors

Cancer survivors (hereinafter referred to as patients) were recruited as the primary audience of the fotonovelas. Following the 2019 National Comprehensive Cancer Network breast cancer screening guidelines, participants were eligible if they either (1) had been diagnosed with ovarian cancer at any age or (2) had been diagnosed with breast cancer at age 50 or younger or (3) had been diagnosed with triple-negative breast cancer at age 60 or younger or (4) had been diagnosed with breast cancer at any age and had one or more family member diagnosed with breast cancer before age 50, or (5) ovarian cancer, or (6) male breast cancer, or (7) pancreatic cancer. In addition, to be eligible, participants had to be 18 years of age or older, be able to read in Spanish or English, and have no prior participation in GCT.

#### Relatives of cancer patients

Family members of cancer patients were also recruited as a target audience of the fotonovelas. To be eligible for the study as family members, participants needed to (1) have a family history of breast or ovarian cancer, (2) could not have participated in GCT, and (3) could not have been diagnosed with breast or ovarian cancer. All participants in the role of family members were relatives of the participants in the role of patients, were 18 years of age or older, and were able to read in Spanish or English.

#### Health workers

Health workers (HWs) were recruited to be part of the study as knowledge experts of the patient/provider interaction in the topic of GCT to provide feedback on the content of the fotonovelas. Therefore, the eligibility criteria for health workers were about their work experience and knowledge of GCT. To be eligible to participate in the study, participants had to (1) be working as health workers for over a year, (2) worked with Latina patients with breast or ovarian cancer, and (3) reported having some knowledge of GCT (self-rated knowledge). Health workers also had to be at least 18 years of age and able to read in Spanish or English.

### Recruitment process

Eligibility screeners were developed in English and translated into Spanish for each participant group/role (Appendix 2). The screeners included a brief description of the study and a series of questions for the recruitment team to ask potential participants to determine eligibility. The participant recruitment was led by the partnering community-based organization, which provides navigation and support to Latina women with cancer. A flyer was also developed by the study team in English and Spanish. The staff at the community-based organization shared the flyer at community meetings and called families they had worked with in the past to inform them of the study. Individuals who were interested in the study were screened for eligibility over the phone (i.e., screening call). Additionally, participants’ self-reported age and sex were collected during the screening call. After the screening calls, all interviews were done in-person at various locations. Participation in the interviews was voluntary, and informed consent was provided verbally by phone before the interviews.

#### Interview guide

The study team developed interview guides to facilitate the semi-structured interviews. The interview guides for patients and relatives included quantitative scales for willingness to discuss cancer with the family and perceived knowledge of GCT (e.g., self-rated knowledge). The interview guide for health workers did not include questions about willingness to discuss cancer with family or knowledge of GCT. All the interview guides also included open-ended questions about the fotonovela (e.g., initial reactions, feedback, and fotonovela comparative questions/narrative preference), the questions are available in Appendix 3.

#### Quantitative scales - sociodemographic factors

Sociodemographic data collected were self-reported age (in years) and sex (male or female). In addition, language preference was measured as the language the participant spoke most often at home (I always speak Spanish, I speak more Spanish than English, I speak Spanish as often as I speak English, I speak more English than Spanish, or I always speak English).

#### Quantitative scales - primary outcomes

Cancer patients and their relatives were asked two items to assess pre-post differences in (1) their willingness to discuss cancer with their family using a 5-point Likert scale (1 = very unlikely, 2 = unlikely, 3 = neutral, 4 = likely, 5 = very likely), and (2) whether they had heard of genetic counseling or testing (yes or no). If yes, participants were then asked to self-rate their knowledge of GCT (1 = no knowledge, 2 = some knowledge, 3 = very knowledgeable). The participants that reported never hearing of GCT before, were reported as having no knowledge of GCT. These two items were asked again after the participants had read the fotonovelas. Patients and relatives also completed an additional item post-fotonovela readings, inquiring about their likelihood of seeking additional information on GCT, and this was measured using a 5-point Likert scale (1 = very unlikely, 2 = unlikely, 3 = neutral, 4 = likely, 5 = very likely).

#### Qualitative data

The open-ended questions focused on the participants’ initial reaction, main idea recall, tone, acceptability/initial thoughts, level of engagement/memorability, format, and intention of sharing the information learned (e.g., “What are your initial reactions and thoughts on the fotonovela?”, “What was the main idea the fotonovela was trying to convey?”, etc.). In addition, participants were asked fotonvela comparative questions. For example, “Which fotonovela was the most memorable one?”, “Which fotonovela would you share with a friend or family member?”, etc.

### Participant interviews and procedures

Two members of the study team (R.B. and C.B.) underwent moderator training led by *ICF International* in preparation for the interviews. These researchers, and occasionally a trained volunteer, conducted in-depth interviews from February to March 2019. All interviews were conducted in person and in a variety of locations, depending on the participant’s preference, including a nearby church, a public library, or the office of the community organization that assisted with recruitment. The interviews were conducted in Spanish or English, depending on the preference of the participant. The average length of the interviews was 45 min (ranging from 30 min to one hour). All participants received a $75 gift card.

All participants conducted individual interviews. After introductions, interviewers read out loud a brief description of the study and explained the purpose of the interview. Interviewers mentioned that all responses were voluntary. Participants were then asked if they agreed to have the interview audio-recorded, with the caveat that their names would not appear on the recording. All participants agreed to have the interview recorded. Once the recording began, the interviewer stated the participants’ identification number, the date of the interview, and the time. Following an “emotional greeting”, where the participants and the interviewer shared something about themselves (e.g., their favorite food, a hobby, etc.), the interviewers began with the quantitative scales. Afterward, participants were given one fotonovela at a time to read. The order of the fotonovelas was random. Once the participant finished reading the first fotonovela, the interviewers proceeded with open-ended questions (e.g. fotonovela-feedback questions).

### Data analysis

#### Data analysis for quantitative data

Quantitative data, including sample demographics and responses for the pre-and post-fotonovela questions, were entered into the Stata MP 17 program. Frequencies were computed to obtain descriptive statistics and the McNemar’s test was used to examine differences in proportions of the outcomes pre-and post-fotonovelas. The level of statistical significance was set at an alpha of 0.05. To increase power in the quantitative analyses, some response categories for the questions were grouped. Specifically, (1) the status of openness to discussing cancer with their family was categorized as unlikely (including very unlikely, unlikely, and neutral answers) or likely (including likely and very likely answers); (2) knowledge of GCT was categorized as no knowledge and knowledgeable (including some knowledge and very knowledgeable). Additionally, descriptive frequencies were obtained regarding the participants’ willingness to seek further information on GCT post-fotonovelas, measured as unlikely (including very unlikely, unlikely, or neutral) or likely (including likely and very likely).

#### Data analysis for qualitative data

The company ICF International transcribed the interview recordings verbatim and translated them into English, providing copies of the transcripts in both English and Spanish. Interview transcripts were reviewed after all interviews were completed. Data saturation was not assessed during the course of the interviews. To develop the codebook, three members of the study team (C.B., S.S., A.H.) independently reviewed a selected transcript from each of the participant groups to identify initial themes. These themes were then shared in a meeting with additional research partners for input and refinement (S.G.). Next, two members of the study (C.B. and S.S.) reviewed the remaining transcripts in the original language (Spanish) to identify additional themes. The themes revolved around the interview guides, which focused on knowledge of GCT and suggestions for improving the fotonovelas. The selected deductive codes were (1) the purpose and impact of the fotonovelas, (2) acceptability/initial thoughts of the fotonovelas, (3) intentions regarding GCT after reading the fotonovelas (e.g. sharing information learned), (4) suggestions for improving the fotonovelas, and (5) new information learned from the fotonovelas. An additional inductive code emerged from the interviews and was added to the codebook: (6) perceived barriers to GCT. The team agreed on the final set of codes for which parent and child codes were developed and entered into Dedoose qualitative software. Two researchers (C.B. and S.S.) independently coded the transcripts that were in Spanish and met weekly to reconcile and refine the codes as needed. Following the consensual qualitative analysis principles (Hill, Thompson, and Williams 1997), any coding discrepancies were discussed until a consensus was reached. Once all transcripts were coded, two researchers (C.B. and Y.R.) summarized the results according to participant groups.

## Results

A total of 30 participants were interviewed, including 10 Latina cancer patients, 10 family members of cancer patients, and 10 health workers. However, the audio recording of two interviews with family members was not fully captured, leading to a final study sample of *n* = 28. The sample demographic characteristics are found in Table 1. Across all participants, 43% of participants reported speaking more Spanish than English at home, and nearly all interviews were conducted in Spanish (except one). All patients were female, and their ages ranged from 33 to 60 years (M = 51, SD = 9). Among relatives, their ages ranged from 20 to 58 years (M = 40, SD = 12). As for HWs, their age ranged from 24 to 59 years (M = 44, SD = 11) and their years of experience working as a health worker ranged from 4 to 30 years (M = 12, SD = 9).

**Table 1:** Sample demographic characteristics of the Fotonovela Project participants, including Latina cancer patients, relatives of cancer patients, and health workers

Characteristics	Patient *n* = 10	Relative *n* = 8	Health Worker *n* = 10	Total N = 28
**Fotonovela Familiarity with Fotonovelas**				
Yes	8 (80)	4 (50)	8 (80)	20 (71)
No	2 (20)	4 (50)	2 (20)	8 (29)
**Familiarity with genetic counseling and testing**				
Yes	6 (60)	6 (75)	8 (80)	20 (71)
No	4 (40)	2 (25)	2 (20)	8 (29)
**Years of work experience**, mean (SD)*	N/A	N/A	12 (9)	N/A
**Language preference**				
I always speak Spanish	4 (40)	3 (37.5)	1 (10)	8 (29)
I speak more Spanish than English	6 (60)	3 (37.5)	3 (30)	12 (43)
I speak Spanish as often as I speak English	0	2 (25)	3 (30)	5 (18)
I speak more English than Spanish	0	0	2 (20)	2 (7)
Not recorded	0	0	1 (10)	1 (4)
**Sex**				
Female	10 (100)	6 (75)	8 (80)	24 (86)
Male	0	2 (25)	2 (20)	4 (14)
**Age, mean (SD)**	51 (9)	40 (12)	44 (11)	45 (13)

*Notes* Percent reported in parentheses. Not applicable (N/A). *Question only applied to health workers

### Quantitative outcomes

**Fig. 1:**
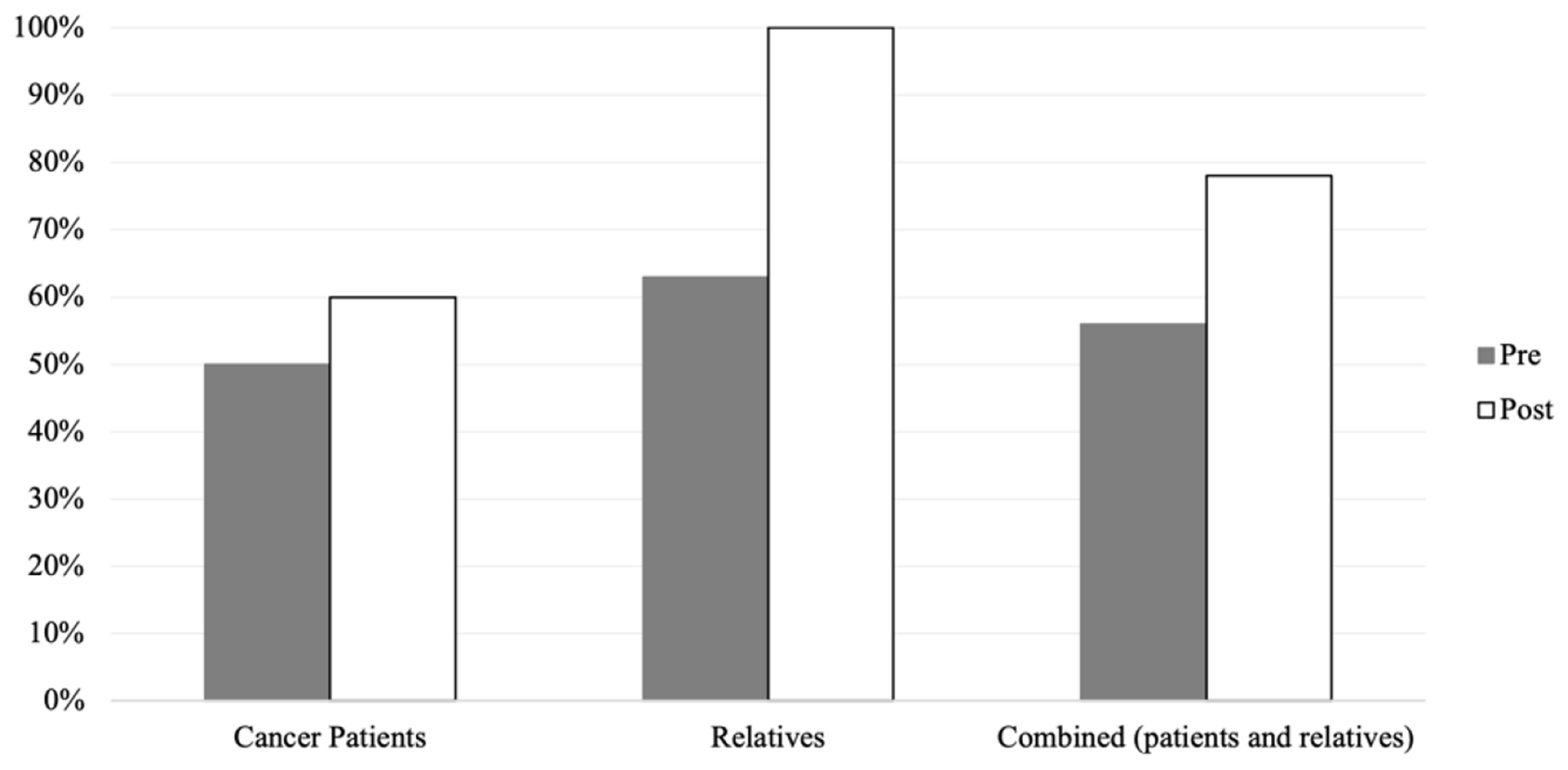
Knowledge of genetic counseling and testing pre-and post fotonovels

**Fig. 2:**
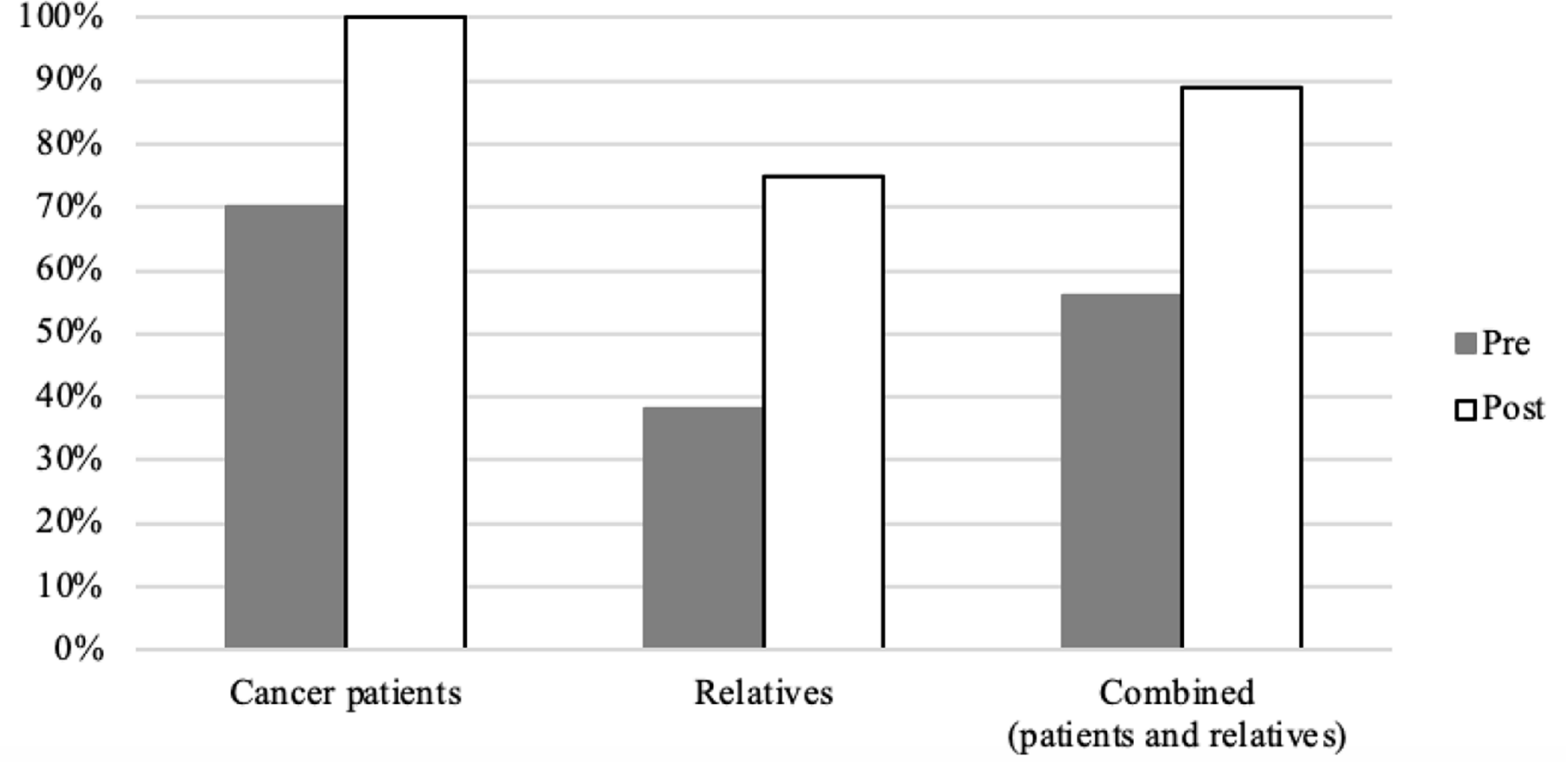
Likelihood of disussing cancer with family pre-and post fotonovels

Descriptive frequencies of the quantitative outcomes are found in Table 2. Reading the fotonovelas increased self-rated knowledge of GCT in cancer patients from 50 to 60% (10%) and in family members from 63 to 100% (37%), with an overall increase of 22% (Fig. 1), however, this finding was not statistically significant (p-value = 0.16). Similarly, reading the fotonovela increased willingness to talk about cancer with family from 70 to 100% in cancer patients (30%) and from 38 to 75% in family members (37%) (Fig. 2), with an overall statistically significant increase of 33% (p-value = 0.02). Additionally, 100% of the patients and relatives reported being likely to seek more information about GCT after reading the fotonovelas.

**Table 2:** Descriptive frequencies of pre-and post-fotonovela question responses among Latina cancer patients at risk of Hereditary breast and ovarian Cancer, relatives of cancer patients, and health workers

Measures		Patient *n* = 10		Relative *n* = 8	
		Pre	Post	Pre	Post
**Knowledge of genetic counseling and testing**					
No knowledge		5 (50)	4 (40)	3 (38)	0
Some knowledge / very knowledgeable		5 (50)	6 (60)	5 (63)	8 (100)
**Willingness to discuss cancer with family**					
Very unlikely/unlikely/neutral	3 (30)		0	5 (63)	2 (25)
Likely/very likely		7 (70)	10 (100)	3 (38)	6 (75)

### Fotonovela narrative preference

Results from the fotonovela comparison questions are found in Table 3. Across all participant groups, the most memorable fotonovela was the one with the family narrative. When cancer patients were asked which fotonovela they would share with a family member or friend, half of the patients (50%) stated that they would share the *family fotonovela*. Whereas, among family members, there was a tie between the *friend fotonovela* and the *doctor fotonovela* (both selected by 37.5%). Among health workers, there was also a tie between the *friend fotonovela* and the *doctor fotonovela* (both selected by 30%). Furthermore, when asked which fotonovela would make readers most interested in learning more information about GCT, half of the patients (50%) and most family members (75%) selected the *doctor fotonovela*. Whereas most of the health workers (40%) selected the *family fotonovela* as the one that would motivate participants to seek more information.

**Table 3:** Descriptive frequencies of fotonovela- comparative question responses from Latina cancer patients at risk of hereditary breast and ovarian cancer, relatives of cancer patients, and health workers

Measures	Patient (*n* = 10)		Relative (*n* = 8)		Health Worker (*n* = 10)		Total (*n* = 28)
**Most memorable fotonovela**							
Family	7 (70)	4 (50)		4 (40)		15 (54)	
Friend	0	1 (12.5)		2 (20)		3 (11)	
Doctor	3 (30)	2 (25)		3 (30)		8 (29)	
No response	0	1 (12.5)		1 (10)		2 (7)	
**Preferred fotonovela to share with a friend or family member**							
Family	5 (50)	2 (25)		2 (20)		9 (32)	
Friend	3 (30)	3 (37.5)		3 (30)		9 (32)	
Doctor	1 (10)	3 (37.5)		3 (30)		7 (25)	
No response	1 (10)	0		2 (20)		3 (10)	
**The fotonovela that made the participants most interested in learning more about genetic counseling and testing** ^a^							
Family	1 (10)	1 (12.5)		4 (40)		6 (21)	
Friend	3 (30)	1 (12.5)		0		4 (14)	
Doctor	5 (50)	6 (75)		3 (30)		14 (50)	
No response	1 (10)	0		3 (30)		4 (14)	

^a^The question was rephrased for health workers as “Which fotonovela do you think would make a patient more interested in learning more about genetic/testing counseling.”

### Qualitative themes

#### Fotonovela purpose: remembering the main idea

Across all groups, participants recognized the primary goal of the fotonovelas, which was to increase knowledge of GCT. While the overall focus was on GCT (e.g., how it is performed, how one can receive genetic counseling to see if they should undergo genetic testing, and the meaning of a positive and negative genetic test result), the educational content also briefly covered HBOC and encouraged readers to be aware of their family history of cancer. Moreover, the majority of participants (75%) commented that the message of the fotonovelas was to inform readers of GCT and its importance (e.g., understanding one’s risk of HBOC to make informed decisions), including six patients (60%), six family members (75%), and nine health workers (90%) (selected quotes are shared in Table 4). Additionally, two patients (20%) and two health workers (20%) expressed that an important message of the fotonovela was to take preventive measures in the event of a possible future cancer diagnosis. Three patients (30%) also mentioned that an important component of the fotonovelas was the clarification between cancer genetic testing and cancer diagnostic testing, and the meaning of a negative result. For example, a patient commented, *“So*,* this is good because he (the doctor) is saying that even if she (the patient) comes out negative (referring to the genetic test result)*,* her children are not out of risk (of being diagnosed with cancer in the future)”* (P-4). Additionally, two health workers (20%) expressed the importance of sharing cancer-related stories with family members to encourage coping with difficult situations together.

**Table 4:** Qualitative themes from the Fotonvela Project on genetic counseling and testing

Themes	Quotes		
	Patients	Relatives	Health Workers
Fotonovela purpose: remembering the main idea	“The direct message is about genetics, and I think it’s… it’s well the message” (P-6). “Amm… the fact of awakening that… in people the curiosity to have that genetic test, to know more about it… and also as a preventative way… Or let’s say as prior information not… not so much preventive, because even if you know, you don’t prevent it but, but as a way to take actions before” (P-38). “The importance of getting together, so that she [the patient] can pass on information to the whole family because here [in the community] that is rarely the case…I like it because they [the characters] explain that if the test comes back negative, it’s not that they [the patients] are safe from cancer, but like anyone else, and it explains that it doesn’t hurt [the procedure] because that’s the first thing they ask” (P-39).	“The message that I think that the fotonovela conveys is that people should be more informed about genetic testing” (R-16). “Amm… how easy the genetic testing would be” (R-20). “The fotonovela wants to raise awareness and to talk to one’s children about it, whether it is any type of cancer, and to have a genetic test to see if they [the family/patients] have a high risk of having cancer or how sometimes the genetic test is not that it detects if they have cancer but the risk” (R-34).	“That it’s not painful [genetic test procedure], well it’s just, ah… it’s a saliva test” (HW-1). “This is a message from a woman who went through cancer and is trying to educate other people, saying, you have to do it because it’s important” (HW-2). “The message has been information and prevention” (HW-29).
Acceptability/ Credibility/ Initial Thoughts	“I think it’s super, super good because it really explains what genetics is all about…and that, no, it’s not a diagnostic test” (P-7). “Excellent because… it’s the communication between mother and children, explaining what can happen” (P-16). “I don’t know what word to use, like quite hmm… I don’t know if it would be honorable or thoughtful that this is done for Hispanics” (P-38)	“Excellent, this makes us [the readers] seek more information and be aware of what might happen” (R-16). “This one is more like…it keeps you paying attention a little bit more” [comparing the fotonovelas to a brochure] (R-20). “I think it is easy to believe, to understand” (R-31).	“Excellent, you have to use it” (e.g., using this tool to educate Latinos about genetic counseling) (HW-1). “This is something very important” (HW-32). “I like it [the fotonovelas], it has a lot of detail, and I think that it is important…I think it could be a good tool because it is… amm… simple to understand” (HW-37).
New information learned from the fotonovelas	“That if the test is positive, if it means you already have cancer… and then they say no, right? That they only see the risk” (e.g., understanding that a positive result in a genetic test does not mean the patient already has cancer) (P-6). “I didn’t know that diseases also came genetically” (P-11). “The importance of passing it on to the family” (e.g., family history of cancer) (P-39).	“That a test of saliva or blood can be used” (R-19). “That no matter if it is positive or negative [referring to the genetic test] if we had a family member who has had cancer, we can still have a chance that we may have cancer even if it was negative [the genetic test result]” (R-40). “Knowing how the test is performed …makes you lose your fear of the test” [referring to the genetic test] (R-16).	Not applicable (health workers were not asked about new information learned)
Intentions /actions after reading the fotonovelas (e.g. sharing information learned or seeking more information)	“Well, if I have a friend, I will call her and tell her what I have read. And that it is important to her” (P-4). “Try to find a way to do the test and share it with more people, friends, my children, my family, etc.” (P-42). “Discuss it with, with my friends, with my family, with, mostly with my daughter, it’s true” [discussing the information on the fotonovelas] (P-6).	“Call the doctor and do the test” [genetic test] (R-16 & R-17). “They [the readers] would try to investigate further” (R-31). “If I had read this [the fotonovela] at home, well, if I was with my wife… my children… I would talk about it, I would comment…and try to talk a little bit more about cancer” (R-34).	“I think they [the patient/reader] might want to see a genetic counselor” (HW-30). “Probably look for more information on the test [genetic test] or talk to a counselor or a doctor” (HW-37). “If I am a cancer patient, I would want to become aware of my disease…and I think I should go and talk to my family, to pass this information on” (HW-27).
Suggestions for improving the fotonovelas	“Maybe adding a little bit more about ovarian cancer” (P-39). “She [the mother] shouldn’t talk to them [the children] with fear, she should talk to them differently…like, look, this is not for you to be afraid” (P-41).	“Discussing colon for men, prostate as well, all that” (R-43). “The other thing would be being able to listen to this. That’s the only change there could be, a video, a recording” (R-19).	“Clarifying also that the fact that you are negative does not mean that you are safe forever” [referring to a negative result in a genetic test] (HW-1). “Who are candidates and who are not? It is like clearing up doubts about who should be tested or not” [referring to who should undergo genetic testing] (HW-26). “Put something positive: “He gives the hardest battles to the strongest warriors”, change it a little bit here, the sentence, God always gives the chance” (HW-27).
Perceived barriers to genetic counseling and testing	“Sometimes they [patients/people] say, “we don’t have money”… they don’t know where there may be clinics where they do it for free to those with low benefits.” (P-42) “Among the family, we almost never talk about it [cancer]… but I think it happens with a lot of people too….I think something like fear, I don’t know, due to respect or remembering… what happened (P-9).	“I tell you one thing: I also know that many people do not pay for insurance because it is too expensive, so people who work, work and sometimes do not worry about their health, just work, work” (R-34). “Well, I admit I wish my daughters didn’t know [about family history of cancer]…. because… the suffering is for the family after all” (e.g., highlighting challenges in discussing cancer) (R-19).	“It is very expensive to approach a counselor, and people still do not know what a counselor is” (HW-26). “Not all insurance companies cover genetic testing, it even happened to me, twice that they denied it” (HW-32). “A lot of people think that “Oh, I had a pap smear, and that’s why I know I don’t have ovarian cancer” … they [patients] think that a pap smear can see everything, the whole gynecological part of a woman…well that’s the mistake that a lot of people have” (e.g., highlighting misunderstanding of tests) (HW-2).

*Abbreviations* GCT, genetic counseling and testing; P, patient; R, relative; HW, health worker

#### Acceptability/ initial thoughts

Among all groups, participants commented that the fotonovelas were important and effective in conveying the value of GCT in the Latino community. Two patients (20%) mentioned that the fotonovelas showed meaningful emotional expressions between patients and family members, which they liked. For example, one patient commented, *“I liked it because everything is explained…even the expressions in the fotonovela*,* you can see the person who is helping someone….the whole story is perfect*” (P-8). In addition, two family members (25%) mentioned that the fotonovelas were a good tool for sharing information (as opposed to brochures). Patients and family members also seemed to connect with the story. For example, one family member commented that she identified with the Latina patient (referring to Rosa, the main character) and one patient expressed gratitude for the fotonovela and mentioned that it was an honor that this tool was made for Latinos (e.g., quote from P-38 in Table 4). In addition, several participants (39%) found the fotonovelas easy to understand, including six family members (60%) and five health care workers (50%), as reflected in one patient’s comment, *“It’s quite readable*,* so you understand it and you grasp it quickly”* (P-39).

#### New information learned from the fotonovelas

Regarding new information learned from the fotonovelas, patients reported that they had learned about cancer prevention and its relationship to genetics. Two patients (20%) mentioned that they did not know that a Pap smear was not sufficient to detect certain types of cancer (e.g., beyond cervical cancer). For example, one patient stated, *“Many women think that a Pap smear has to do something with ovaries*,* but they are two different things”* (P-6). In addition, most family members (88%) mentioned learning what a genetic test is and why it is important to get tested. Similarly, three family members (38%) mentioned learning that genetic testing could be done through a blood or saliva sample. In addition, one patient noted that a positive genetic test does not mean that the patient being tested has cancer, but rather informs the patient of his or her risk of developing cancer in the future (e.g., quote from P-6 in Table 4). Although some genetic tests can be diagnostic, for the purposes of the fotonovela, we focused only on assessing the risk of HBOC.

#### Intentions /actions after reading the fotonovelas

When participants were asked what they (or others) would do if they had just read the fotonovela at home, or how they would be affected by the content, most mentioned readers would be motivated to seek more information or would share the information with others. Specifically, three patients (30%) and one family member mentioned they would share the information learned with friends and their children, particularly their daughters (e.g. quotes from P-4, P-42, P-6, and R-34 in Table 4). In addition, two patients (20%) indicated they would seek additional information about genetic testing services, while two family members (25%) mentioned they would be motivated to get genetic counseling and to visit a doctor (e.g., quotes from HW-30 and HW-27 in Table 4). Additionally two other family members (25%) mentioned that readers would probably feel concerned after reading the fotonovelas, especially if they knew of relatives who had suffered from cancer. Similarly, five health workers (50%) mentioned that patients would likely seek additional information about GCT and that the fotonovelas would make participants more cautious about cancer.

#### Suggestions for improving the fotonovelas

Adding new information. Three patients (30%) emphasized the need for additional information on ovarian and cervical cancer and the difference between having a Pap smear and a genetic test. Two patients (20%) and one relative suggested adding contact information for local genetic counselors and testing centers to facilitate access to information: *“It [the fotonovela] only tells you*,* visit a counselor. But where? How?”* (P-7); *“Are there genetic counselors? Where are they?”* (R-17). Three patients (30%) also suggested adding more details in simple terms about cancer, hereditary cancers, family trees and genetics, and genetic testing before and after a cancer diagnosis. Both family members and health workers suggested adding other cancers that affect men, such as prostate and colon cancer, with appropriate resources: *“I would say that to annex another type of cancer as for men”* (R-34). In addition, two health workers (20%) expressed the need to clarify the meaning of a negative result on a genetic test. For example, HW-1 suggested *“clarifying that the fact that you are negative* [referring to a genetic test result] *does not mean that you are safe forever.”* A health worker also recommended explaining more in detail the meaning of a positive result and conveying that knowing the result can be beneficial regardless of whether it comes out positive or negative. As HW-30 noted, *“like a little bit more about what happens when*,* if it’s negative and if it’s positive…and that no matter what the result is*,* the result can always help”* (e.g., regardless of the result, it can guide next steps).

Format. Two patients (20%) and three health workers (30%) suggested bigger fonts and adding more color to the fotonovelas. Three family members (37.5%) suggested making the fotonovelas available in a recording or a movie; “*The fotonovela is beautiful*,* but if it were in a video*,* it would be better*” (R-17). Similarly, two health workers (20%) suggested creating an animated version to make the characters more expressive and vibrant. Another suggestion from four health workers (40%) was to use figures to facilitate understanding of the content and reduce text. Furthermore, three health workers (30%) recommended shortening the three versions of the fotonovelas.

Characters/ conversation tone. Overall, participants recommended building the relationships between the characters more to make the story more realistic. A patient recommended being more inclusive regarding the ages, although, it was unclear if this referred to the age of genetic testing recommendation. Another recommendation was to base the narrative of the fotonovela on a true story where readers could identify themselves or their relatives. For example, P-30 commented *“As for it to be more credible … when people look at it*,* they say: “Oh*,* yes” it can happen in real life.”* Additionally, two patients (20%) felt that the conversations between the characters were too direct or unemotional and highlighted the importance of transmitting trust through the fotonovela, especially between the patient and doctor: *“You have to gain the trust of the patient… the person comes in*,* and they want to hear this—that they explain a little bit of what it is (genetic test)*,* what it’s about*,* what it’s going to be like.”* (P-7) (e.g., suggesting improved communication between the patient and doctor).

Two family members (25%) and a health worker recommended changes in the tone of the fotonovela to make it more realistic: *“The real situation would not have been that kind of tone; it would have been more dramatic”* (R-31); *“It is intimidating … it lacks a little relaxation in the family”* (HW-32). Four health workers (40%) also commented on how the conversations between the characters in general seemed very tense (unnatural). Three health workers (30%) suggested making the facial expressions of the doctor less serious and more compassionate. For example, *“But I feel that I don’t know*,* the person is giving the message with a very worried face”* (HW-2) and *“That the doctor could show a little more compassion”* (HW-28). It was also recommended that the fotonovela incorporate action steps into the conversations. For example, after the doctor tells the patient that she needs to make an appointment, the patient can say *“Okay*,* thank you*,* let’s make the appointment”* (HW-3). Another recommendation by a health worker was to clarify the type of doctor the patient talks to because, at one point in the fotonovela, the doctor suggests to the patient to go see a genetic counselor, but he is a doctor informing the patient of genetic counseling, so this could confuse readers.

#### Perceived barriers to genetic counseling and testing

Significant barriers to GCT among Latinas were mentioned by participants. Two of the main concerns, especially commented on among health workers, were lack of health insurance and communication with health care providers, including lack of knowledge about genetic testing or difficulty getting referrals for genetic counseling. For example, a health worker describes how expensive health insurance is: *“It was almost the whole salary to pay for insurance*,* very expensive”* (HW-26). A similar sentiment was shared by a family member: *“I tell you one thing: I also know that many people do not pay for insurance because it is too expensive*,* so people who work*,* sometimes do not worry about their health*,* just work and work”* (R-34). A patient also discussed the lack of insurance as a barrier to health care access: *“Unfortunately*,* sometimes we don’t have health insurance…that prevents people from going for checkups”* (P-42). Another barrier to GCT mentioned by patients and family members was fear associated with cancer: *“Among us Hispanics*,* there is this fear of not wanting to go to the doctor”* (R-16). Similarly, a patient discussed the fear associated with doctor visits: *“There are many people who don’t know*,* other people who are afraid to go to the doctor”* (P-41). An additional barrier to GCT was the lack of awareness and education on the topic, as commented by a patient: *“We come*,* many people*,* from countries that maybe… who haven’t had much education”* (P-8). Another patient also discussed the lack of conversations about cancer among families as a barrier: *“Many people do not know this and think that they are the only ones who get cancer and that perhaps they cannot pass it on to other people*,* to their children”* (P-39).

## Discussion

This formative pilot study on the use of fotonovelas to raise awareness of GCT among Latina women at risk of HBOC and their families found fotonovelas to be a promising educational tool. Preliminary findings indicated an increase in willingness to discuss cancer with family after reading the fotonovelas. An increase in self-rated knowledge of GCT was also found after reading the fotonovelas; however, these results would need to be confirmed in a larger study.

Through interviews with participants, we were able to better understand their willingness to discuss cancer with family and the new information learned from the fotonovelas. For example, when asked about their intentions to share the information from the fotonovela, some participants mentioned that they would likely call a friend after reading the fotonovela to share the information learned, while others expressed that the fotonovela would motivate them to talk about cancer with their family, especially their daughters. In terms of new information learned, one of the most common responses was learning about the simplicity of genetic testing (e.g., being able to use saliva). In addition, all participants mentioned that the fotonovelas would be memorable, with the family fotonovela being the most memorable. Participants noted the importance of GCT and the need to share the information with their friends and family as the most memorable aspects of the fotonovelas. These results support previous studies indicating that providing health education in a narrative format to the Latino community, such as with fotonovelas, is an effective approach to help readers remember the content and be willing to share it with others (Pierron 2022; Unger et al. 2013; Chan et al. 2015).

Additionally, comments on the format and presentation of information suggested an interest in learning more about other types of cancer and in making the fotonovela more inclusive of men. While the primary audience for this fotonovela was Latina women at risk of HBOC, given the link between HBOC with increased pancreatic and prostate cancer risk, (Solomon et al. 2012; Giri et al. 2016) it would be important for future educational materials to expand the population to include men. Participants also suggested other formats for sharing information about GCT, including creating a video and using a real-life story as the basis for the fotonovela. While a video on this topic already exists (Hurtado-de-Mendoza et al. 2019, 2020), a fotonovela based on one or more people in the community who have been diagnosed with HBOC and gone through the GCT process could amplify its impact. Participants also suggested making the dialogue between family members and between doctor and patient more relaxed. These suggestions could be incorporated into an animated version of the fotonovela to help readers better relate to the family conversations and also to emphasize the importance of the doctor spending enough time with the patient.

Participants also discussed perceived barriers to GCT among Latinas, especially the tendency to avoid undergoing cancer-related testing due to fear of the diagnosis and unfamiliarity with the procedure. These individual-level barriers, including fear and anxiety associated with cancer among Latinas have also been documented in previous studies (Gómez-Trillos et al. 2019; Hurtado-de-Mendoza et al. 2019; 2018). In addition, participants expressed difficulty in getting referrals for GCT as a barrier. This health care barrier has also been documented by Jagsi et al. (2015), who found that providers were less likely to discuss GCT with minoritized populations, including Latina women, despite their high interest in these services (Jagsi et al. 2015). Another study found that Latinas would undergo GCT if offered by their health care provider, especially once they have a clear understanding of the benefits of GCT (Rajpal et al. 2017). These findings further emphasize the importance of understanding the multilevel barriers to GCT and how patients and providers can advocate for access to GCT.

### Strengths and limitations

To our knowledge, this pilot study is the first to develop and assess Spanish-language fotonovelas focused on increasing GCT awareness among Latina women at risk for HBOC. These fotonovelas contribute to the few culturally targeted educational materials on GCT for Latino families (Conley et al. 2021; Hurtado-de-Mendoza et al. 2020; Joseph et al. 2010; Sussner et al. 2010). Another strength of this study was obtaining feedback on the fotonovelas from three perspectives: cancer patients, their relatives, and health workers, which is not often achieved (Shete et al. 2023). In addition, community partners provided input in the selection of the fotonovelas. Recent studies have highlighted the benefits of a “co-design” model, in which community members and health professionals are considered “co-experts” and participate equally in the development of educational tools to raise awareness of GCT (Shete et al. 2023; Ali et al. 2019).

Moreover, this study also comes with limitations. First, the small sample size (*n* = 28) was a limitation for performing statistical analyses. Thus, no strong claims can be made in terms of statistically significant differences from the pre-and-post fotonovela questions, although preliminary findings are promising. Second, the outcomes were assessed with single items (i.e., Likert-style scale), however, through the qualitative interviews, we were able to gather further insights into the outcomes (such as new information learned and intentions of sharing the information). Third, GCT knowledge was self-rated and longer term retention testing was not feasible with the available funding. Fourth, the results of the study may not be generalizable, given that we partnered with a community-based organization that has been connecting the Latino community with GCT services for years. Therefore, participants in this study may be more knowledgeable about GCT compared to others. Fifth, the fotonovelas were primarily piloted in Spanish (only one interview was in English), as most participants spoke Spanish more than English. Therefore, we are not sure if the fotonovelas would be applicable to English-only-speaking Latinas. Sixth, this study measured perceived intentions after reading the fotonovelas (e.g., sharing the information learned or seeking more information on the topic), however, post-fotonovela behaviors such as obtaining GCT were not measured. Future studies should evaluate these behavioral outcomes.

### Practice implications

Health centers should be equipped with culturally appropriate educational tools to improve patients’ health literacy. This pilot study points to fotonovelas as a potential tool to help increase knowledge about GCT among Latina women at risk for HBOC and encourage having cancer-related conversations among family members. In addition, given the high prevalence of breast cancer among Latina women, it is critical to continue to find ways to reduce the multilevel barriers to GCT. Health care providers should be aware of the options available to patients that can facilitate their access to GCT, including options for patients who do not have health insurance. Providers should also be mindful of communication barriers among Spanish-speaking patients. Although interpretation services are increasingly offered in healthcare settings, translation does not always facilitate understanding of healthcare topics (Joseph et al. 2023). In addition, the use of fotonovelas could be explored among other racial/ethnic backgrounds and modified to address concerns regarding the barriers to GCT faced by other populations. Future research should explore strategies for implementing the use of fotonovelas in healthcare facilities and other public spaces, as well as alternative approaches to disseminating information about GCT and increasing access to GCT.

## Electronic supplementary material

Below is the link to the electronic supplementary material.

Supplementary Material 1

Supplementary Material 2

Supplementary Material 3

## Data Availability

No datasets were generated or analysed during the current study.
