# Supplementary material for: Raising awareness and education of genetic testing and counseling through fotonovelas among Latina women at risk for hereditary breast and ovarian cancer
Source: J Community Genet. 2024 Sep 6;15(5):475–88. doi: 10.1007/s12687-024-00728-5 (PMC11549277; doi:10.1007/s12687-024-00728-5)
Supplement: Supplementary file 1 — Supplementary Material 1 [file 12687_2024_728_MOESM1_ESM.docx]

**Raising Awareness and Education of Genetic Testing and Counseling through Fotonovelas among Latina women at risk for Hereditary Breast or Ovarian Cancer**

**Journal of Community Genetics**

**Author information**

Rolando Barajas, MPH^1,6^, Clara B. Barajas, MPH^2^, Yaideliz M. Romero Ramos^3^, Sara Gómez Trillos, MS^2,4^, Sabrina Sawhney^2^, Claudia Campos^5^, Alejandra Hurtado-de-Mendoza, PhD^2,4^, Melissa Rotunno, PhD^6^, Elizabeth Gillanders, PhD^6^

1. Georgetown University School of Medicine, Washington, DC, USA
2. Georgetown University, Cancer Prevention and Control, Lombardi Comprehensive Cancer Center, Washington, DC, USA
3. Brown University School of Public Health, Department of Epidemiology, Providence, RI, USA
4. Fischer Center for Hereditary Cancers, Washington, DC, USA
5. Nueva Vida, Inc., Alexandria, VA, USA
6. National Health Institutes/National Cancer Institute, Division of Cancer Control and Population Sciences, Genomic Epidemiology Branch, Bethesda, MD, USA

**Corresponding Author:** Clara B. Barajas, [clara.barajas@georgetown.edu](mailto:clara.barajas@georgetown.edu)

**Funding:** This study was funded by the National Health Institutes/National Cancer Institute, Division of Cancer Control and Population Sciences CRAFT Grant.

**APPENDIX 2 – ELIGIBILITY SCREENERS**

**PATIENT SCREENER**

*Hello, I’m __________ from [name of workplace]. I’m calling today on behalf of the National Cancer Institute, or NCI. The NCI is conducting a research study about how people would react to a Spanish educational material on the benefits of genetic counseling for Latina cancer patients—and would like to include your views. I need to ask you just a few questions to determine whether I can invite you to participate in an in-depth interview. Some of the questions are about your personal experiences with cancer. Your responses will be completely confidential and private and not shared with others. My questions today will only take a few minutes of your day.*

Is this a good time to talk with you a little bit more about the study? ______Yes______No

If **not**: Is there another time that would be better to speak with you? ___________________

1. What is your age? [Numerical answers only.]

- If 17 or under, **TERMINATE.**
- If 18 or over, **CONTINUE.**

2. Do you consider yourself Hispanic or Latina?

|  |  |  | |  |
| --- | --- | --- | --- | --- |
|  | Yes | | 01 | **> CONTINUE** |
|  | No | | 02 | **> TERMINATE** |

3. Have you been diagnosed with Breast or Ovarian cancer?

|  |  |  | |  |
| --- | --- | --- | --- | --- |
|  | Yes | | 01 | **> CONTINUE** |
|  | No | | 02 |  |

4. How old were you when were you when you were diagnosed? [Numerical answers only.]

- Answer________

5. Have you been diagnosed with triple negative breast cancer (estrogen receptor-negative, progesterone receptor-negative, and HER2-negative)?

|  |  |  | |  |
| --- | --- | --- | --- | --- |
|  | Yes | | 01 | **> CONTINUE** |
|  | No | | 02 |  |

6. Do you have a family history of Breast or Ovarian Cancer?

|  |  |  | |  |
| --- | --- | --- | --- | --- |
|  | Yes | | 01 | **> CONTINUE** |
|  | No | | 02 |  |
|  | Don’t know | | 03 |  |

7. If Yes, Who? (relation and side of the family, cancer type, age at diagnosis?)

1. Family member #1
2. Family member #2

8. Have you ever used any services to see if you are at-risk of hereditary breast and ovarian cancer? (done genetic counseling)

|  |  |  | |  |
| --- | --- | --- | --- | --- |
|  | Yes | | 01 | **> TERMINATE** |
|  | No | | 02 | **> CONTINUE** |

9. Can you read in Spanish?

| Yes | 01 | **> CONTINUE** |
| --- | --- | --- |
| No | 03 | **> TERMINATE** |

10. Do you prefer to participate in a Spanish or in an English Language Interview?

| Spanish | 01 | **> CONTINUE** |
| --- | --- | --- |
| No preference | 02 |  |
| English | 03 |  |

11. For this study, we are also interested in talking with family members of breast and/or ovarian cancer patients, to learn more about their experiences. Therefore, there is the opportunity to bring in family members to also be interviewed with you and receive an incentive. Do you have non-cancer family members (daughter, son, husband, etc.) who live in the area, are over the age of 18, and that might be interested in joining the study?

|  |  |  | |  |
| --- | --- | --- | --- | --- |
|  | Yes | | 01 | **> CONTINUE** |
|  | No | | 02 |  |

[ if **yes**, “great I will contact them later and see if they are eligible”]

NOTES FOR RECRUITER:

[Eligibility Decisions]

1. Scenario 1—affected women qualify:
2. Over the age of 18 **AND**
3. They self-identify as Latina/Hispanic **AND**
4. [Have been diagnosed with ovarian cancer OR

diagnosed with breast cancer at age 50 or under OR

diagnosed with triple-negative breast cancer at age 60 or under OR diagnosed with breast cancer at any age and 1 or more relatives with:

- 1. breast cancer before the age of 50 or
  2. ovarian cancer or
  3. male breast cancer or
  4. pancreatic cancer] **AND**

1. They have not participated in genetic counseling or testing **AND**
2. Can read in Spanish.
3. Scenario 2—unaffected women qualify if:
4. Over the age of 18 **AND**
5. They self-identify as Latina/Hispanic **AND**
6. [Have a first or second degree relative:
   1. Diagnosed with breast cancer before the age of 45 **or**
   2. Ovarian cancer **or**
   3. 2 or more individuals with breast cancer primaries on the same side of the family with at least one diagnosed before the age of 50] **AND**
7. They have not participated in genetic counseling or testing **AND**
8. Can read in Spanish.
9. Women are NOT eligible if:
   1. Are under the age of 18 **AND/OR**
   2. They do not self-identify as Latina/Hispanic **AND/OR**
   3. DO NOT meet criteria (“c.”) within both Scenario 1 or Scenario 2 **AND/OR**
   4. Have participated in genetic counseling or testing **AND/OR**
   5. CANNOT read in Spanish.

[IF INELIGIBLE SAY]

1. I want to thank you for your time answering these questions. Unfortunately, you do not qualify to participate in the study. However, please continue to check for opportunities to participate in research in the future. ­­

[SAY IF ELIGIBLE]

1. You are eligible to participate in the project. Participation will include reading through booklets about hereditary breast and ovarian cancer and completing some surveys before and after reading the booklets. We will give you a $75 gift card to thank you for your time. Would you like to participate in the study?
   1. If **No:** May I ask why you are not interested?
      1. Answer:____________________
   2. If **Yes**:
      1. Thank you very much, we will include you in the list of interested participants. Someone from the project team will call you within the next week to give you more information about the study and schedule a day, time, and location for interviews that is convenient for you.
      2. In the next section I will ask you to provide your contact information. Please provide the information to the best of your ability.

**SCHEDULING PROCEDURES**

Have participant provide contact information:

- Full Name:
- Phone Number:
- Best times to contact:
  - Monday_______________________
  - Tuesday______________________
  - Wednesday____________________
  - Thursday______________________
  - Friday________________________
  - Saturday______________________
  - Sunday_______________________

After the participant fills out the contact information:

- Call the participant to schedule the interview.
- If a participant completes the screener and ultimately does not participate in the research for any reason, their data from the screener should be destroyed at the end of the research project.

***END***

**FAMILY RELATIVE SCREENER**

*Hello, I’m __________ from [name of workplace]. I’m calling today on behalf of the National Cancer Institute, or NCI. NCI is conducting a research study about how people would react to a Spanish educational material on the benefits of genetic counseling for Latina cancer patients—as a family member of a Latina BC/OC cancer patient we would like to include your views. I need to ask you just a few questions to determine whether I can invite you to participate in an in-depth interview. Some of the questions are about your personal experiences with cancer within your family. Your responses will be completely confidential and private, they not shared with others. My questions today will only take a few minutes of your day.*

Is this a good time to talk with you a little bit more about the study? ______Yes______No

If **not**: Is there another time that would be better to speak with you? ___________________

1. What is your age? [Numerical answers only.]

- If 17 or under, **TERMINATE.**
- If 18 or over, **CONTINUE.**

2. Do you consider yourself Hispanic or Latino?

|  |  |  | |  |
| --- | --- | --- | --- | --- |
|  | Yes | | 01 | **> CONTINUE** |
|  | No | | 02 | **> TERMINATE** |
|  |  |  |  |  |

3. Have you been diagnosed with Breast or Ovarian cancer

|  |  |  | |  |
| --- | --- | --- | --- | --- |
|  | Yes | | 01 | **> TERMINATE** |
|  | No | | 02 | **> CONTINUE** |

4. Do you have a family history of Breast or Ovarian Cancer?

|  |  |  | |  |
| --- | --- | --- | --- | --- |
|  | Yes | | 01 | **> CONTINUE** |
|  | No | | 02 | **> TERMINATE** |

5. If Yes, Who? (relation and side of the family, cancer type, age at diagnosis?)

1. Family member #1
2. Family member #2

6. Have you ever been a part of any services to see if you or your family are at-risk of hereditary breast and ovarian cancer? (undergone genetic counseling)

|  |  |  | |  |
| --- | --- | --- | --- | --- |
|  | Yes | | 01 | **> TERMINATE** |
|  | No | | 02 | **> CONTINUE** |

7. Can read in Spanish?

| Yes | 01 | **> CONTINUE** |
| --- | --- | --- |
| No | 03 | **> TERMINATE** |

8. Do you prefer to participate in a Spanish or in an English Language Interview?

| Spanish | 01 | **> CONTINUE** |
| --- | --- | --- |
| No preference | 02 |  |
| English | 03 |  |

NOTES FOR RECRUITER:

[Eligibility Decisions]

1. Relatives qualify directly if:
   1. Over the age of 18 **AND**
   2. Self-identify as Latina/Hispanic **AND**
   3. HAVE NOT been diagnosed with Breast/Ovarian cancer **AND**
   4. Have a family history of breast and/or ovarian cancer **AND**
   5. They HAVE NOT participated in genetic counseling or testing **AND**
   6. Can read in Spanish.
2. Relatives are NOT eligible if:
   1. Are not over the age of 18 **AND/OR**
   2. DO NOT self-identify as Latina/Hispanic **AND/OR**
   3. HAVE been diagnosed with Breast/Ovarian cancer **AND**
   4. DO NOT have a family history of breast and/or ovarian cancer **AND/OR**
   5. HAVE participated in genetic counseling or testing **AND/OR**
   6. CANNOT read in Spanish.

[IF INELIGIBLE SAY]

1. I want to thank you for your time answering these questions. Unfortunately, you do not qualify to participate in the study. However, please continue to check for opportunities to participate in research in the future. ­­

[SAY IF ELIGIBLE]

1. You are eligible to participate in the project. Participation will include reading through booklets about hereditary breast and ovarian cancer and completing some surveys before and after reading the booklets. We will give you a $75 gift card to thank you for your time. Would you like to participate in the study?
   1. If **No:** May I ask why you are not interested?
      1. Answer:____________________
   2. If **Yes**:
      1. Thank you very much, we will include you in the list of interested participants. Someone from the project team will call you within the next week to give you more information about the study and schedule a day, time, and location for interviews that is convenient for you.
      2. In the next section I will ask you to provide your contact information. Please provide the information to the best of your ability.

**SCHEDULING PROCEDURES**

Have participant provide contact information:

- Full Name:
- Phone Number:
- Best times to contact:
  - Monday_______________________
  - Tuesday______________________
  - Wednesday____________________
  - Thursday______________________
  - Friday________________________
  - Saturday______________________
  - Sunday_______________________

After the participant fills out the contact information:

- Call the participant to schedule the interview.
- If a participant completes the screener and ultimately does not participate in the research for any reason, their data from the screener should be destroyed at the end of the research project.

***END***

**HEALTH WORKER SCREENER**

*Hello, I’m __________ from [name of workplace]. I’m calling today on behalf of the National Cancer Institute, or NCI. NCI is conducting a research study about how people would react to a Spanish educational material on the benefits of genetic counseling for Latina cancer patients—as a health worker with exposure to Latina BC/OC cancer patients we would like to include your views. I need to ask you just a few questions to determine whether I can invite you to participate in an in-depth interview. Some of the questions are about your personal experiences within your position and cancer patients. Your responses will be completely confidential and private, they not shared with others. My questions today will only take a few minutes of your day.*

Is this a good time to talk with you a little bit more about the study? ______Yes______No

If **not**: Is there another time that would be better to speak with you? ___________________

1. What is your age? [Numerical answers only.]

- If 17 or under, **TERMINATE.**
- If 18 or over, **CONTINUE.**

2. Do you consider yourself Hispanic or Latino?

|  |  |  | |  |
| --- | --- | --- | --- | --- |
|  | Yes | | 01 | **> CONTINUE** |
|  | No | | 02 | **> TERMINATE** |
|  |  |  |  |  |
|  | Refused to answer | | 99 |  |

3. Do you currently work as health worker of some kind: Promotor de salud, patient navigator, community health worker, etc.?)

|  |  |  | |  |
| --- | --- | --- | --- | --- |
|  | Yes | | 01 | **> CONTINUE** |
|  | No | | 02 | **> TERMINATE** |

4. In your line of work do you have contact with Latinas that have been diagnosed with Breast or Ovarian Cancer?

|  |  |  | |  |
| --- | --- | --- | --- | --- |
|  | Yes | | 01 | **> CONTINUE** |
|  | No | | 02 | **> TERMINATE** |

5. How long have you been working as a health worker? [Numerival value]

- If less than a year **[> TERMINATE]**

6. Do you have any knowledge about any services to see if patients are at-risk of hereditary breast and ovarian cancer? (experience with genetic counseling)

|  |  |  | |  |
| --- | --- | --- | --- | --- |
|  | Yes | | 01 | **> CONTINUE** |
|  | No | | 02 | **> TERMINATE** |

7. Do you prefer to read in Spanish or English?

| Spanish | 01 | **> CONTINUE** |
| --- | --- | --- |
| No preference | 02 |  |
| English | 03 | **> TERMINATE** |
| Refused to answer | 99 |  |

8. Do you prefer to participate in a Spanish or in an English Language Interview?

| Spanish | 01 | **> CONTINUE** |
| --- | --- | --- |
| No preference | 02 |  |
| English | 03 | **> TERMINATE** |
| Refused to answer­ | 99 |  |

NOTES FOR RECRUITER:

[Eligibility Decisions]

1. Health workers qualify directly if:
   1. They self-identify as Latina/Hispanic AND
   2. Work as a health worker of some kind AND
   3. Work with Latina BC/OC patients AND
   4. Been in the position for over a year AND
   5. They have knowledge of genetic counseling or testing.
2. Health workers are NOT eligible if:
   1. They do NOT self-identify as Latina/Hispanic AND/OR
   2. Do NOT Work as a health worker of some kind AND/OR
   3. Do NOT Work with Latina BC/OC patients AND/OR
   4. Have NOT been in their position for over a year AND
   5. They do NOT have knowledge of genetic counseling or testing.

[IF INELIGIBLE SAY]

1. I want to thank you for your time answering these questions. Unfortunately, you do not qualify to participate in the study. However, please continue to check for opportunities to participate in research in the future. ­­

[SAY IF ELIGIBLE]

1. You are eligible to participate in the project. Participation will include reading through booklets about hereditary breast and ovarian cancer and completing some surveys before and after reading the booklets. We will give you a $75 gift card to thank you for your time. Would you like to participate in the study?
   1. If **No:** May I ask why you are not interested?
      1. Answer:____________________
   2. If **Yes**:
      1. Thank you very much, we will include you in the list of interested participants. Someone from the project team will call you within the next week to give you more information about the study and schedule a day/time for interviews that is convenient for you. Also, interviews can done at ______________(NCI/Nueva Vida/GT).
      2. In the next section I will ask you to provide your contact information. Please provide the information to the best of your ability.

**SCHEDULING PROCEDURES**

Have participant provide contact information:

- Full Name:
- Phone Number:
- Best times to contact:
  - Monday_______________________
  - Tuesday______________________
  - Wednesday____________________
  - Thursday______________________
  - Friday________________________
  - Saturday______________________
  - Sunday_______________________

After the participant fills out the contact information:

- Call the participant to schedule the interview.
- If a participant completes the screener and ultimately does not participate in the research for any reason, their data from the screener should be destroyed at the end of the research project.

***END***
