# Supplementary material for: Raising awareness and education of genetic testing and counseling through fotonovelas among Latina women at risk for hereditary breast and ovarian cancer
Source: J Community Genet. 2024 Sep 6;15(5):475–88. doi: 10.1007/s12687-024-00728-5 (PMC11549277; doi:10.1007/s12687-024-00728-5)

**Raising Awareness and Education of Genetic Testing and Counseling through Fotonovelas among Latina women at risk for Hereditary Breast or Ovarian Cancer**

**Journal of Community Genetics**

**Author information**

Rolando Barajas, MPH^1,6^, Clara B. Barajas, MPH^2^, Yaideliz M. Romero Ramos^3^, Sara Gómez Trillos, MS^2,4^, Sabrina Sawhney^2^, Claudia Campos^5^, Alejandra Hurtado-de-Mendoza, PhD^2,4^, Melissa Rotunno, PhD^6^, Elizabeth Gillanders, PhD^6^

1. Georgetown University School of Medicine, Washington, DC, USA
2. Georgetown University, Cancer Prevention and Control, Lombardi Comprehensive Cancer Center, Washington, DC, USA
3. Brown University School of Public Health, Department of Epidemiology, Providence, RI, USA
4. Fischer Center for Hereditary Cancers, Washington, DC, USA
5. Nueva Vida, Inc., Alexandria, VA, USA
6. National Health Institutes/National Cancer Institute, Division of Cancer Control and Population Sciences, Genomic Epidemiology Branch, Bethesda, MD, USA

**Corresponding Author:** Clara B. Barajas, [clara.barajas@georgetown.edu](mailto:clara.barajas@georgetown.edu)

**Funding:** This study was funded by the National Health Institutes/National Cancer Institute, Division of Cancer Control and Population Sciences CRAFT Grant.

**APPENDIX 1 – FOTONOVELAS**

1. **Doctor fotonovela – Spanish version**

**
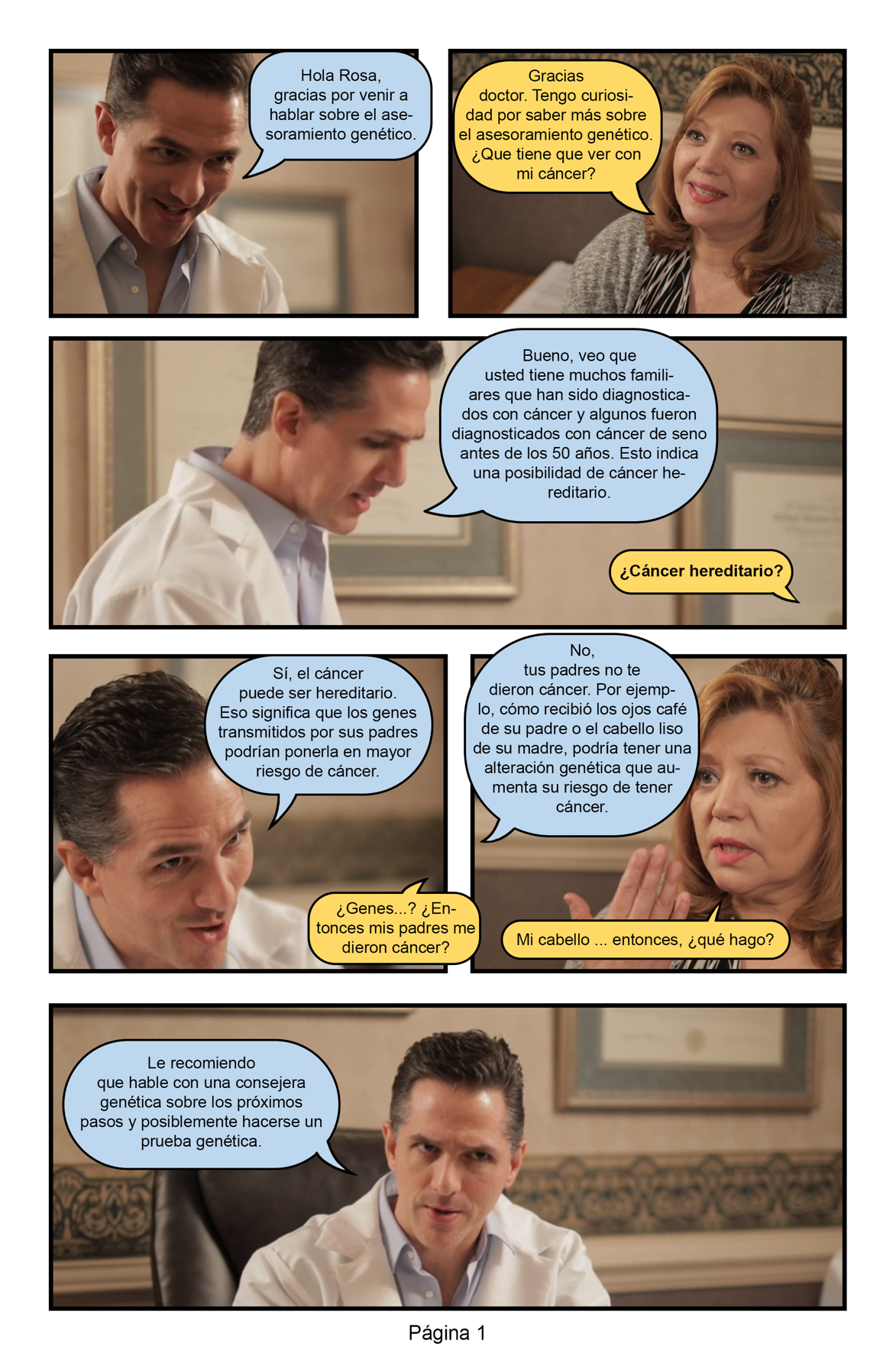
**

**
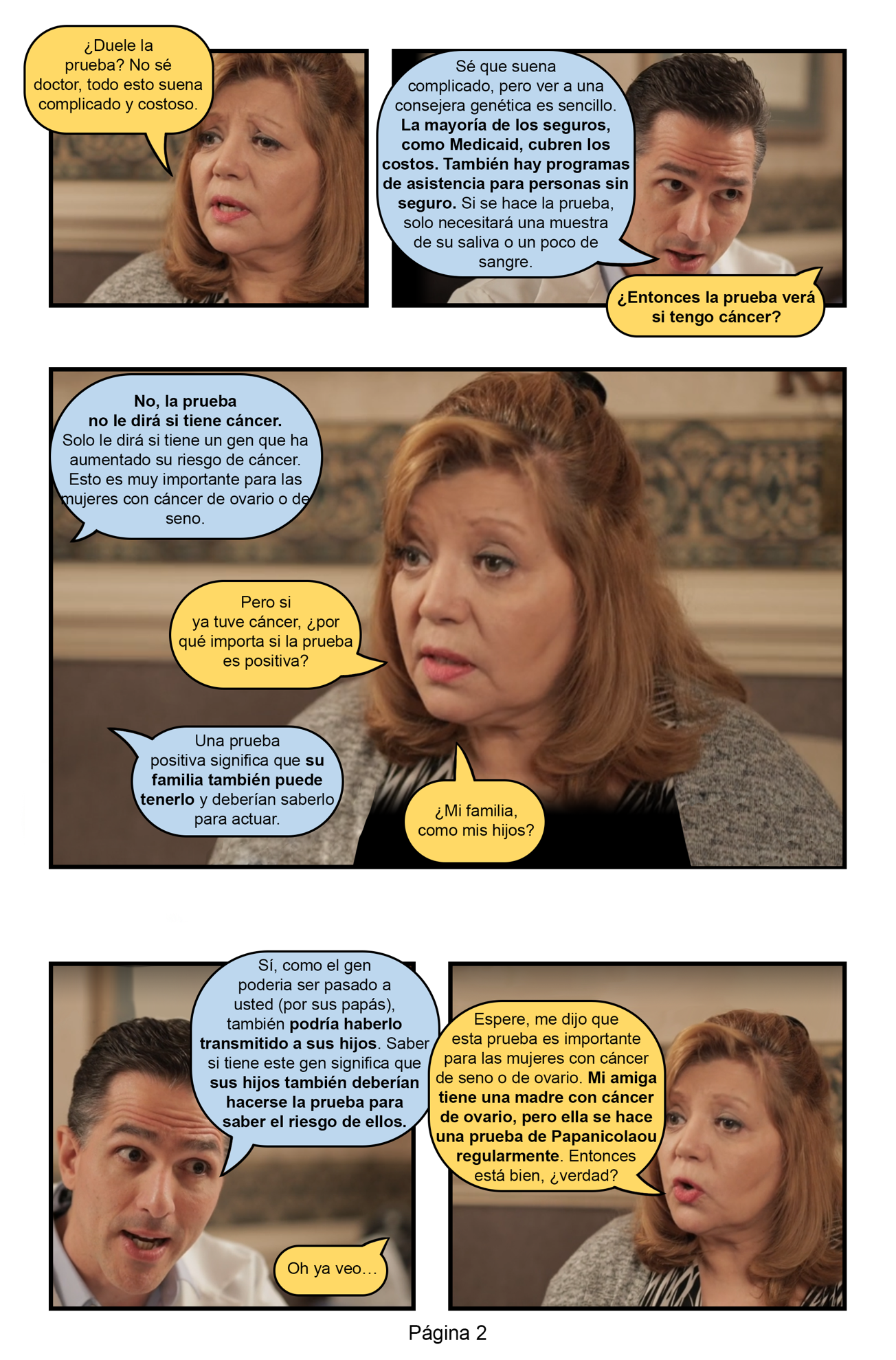
**

**
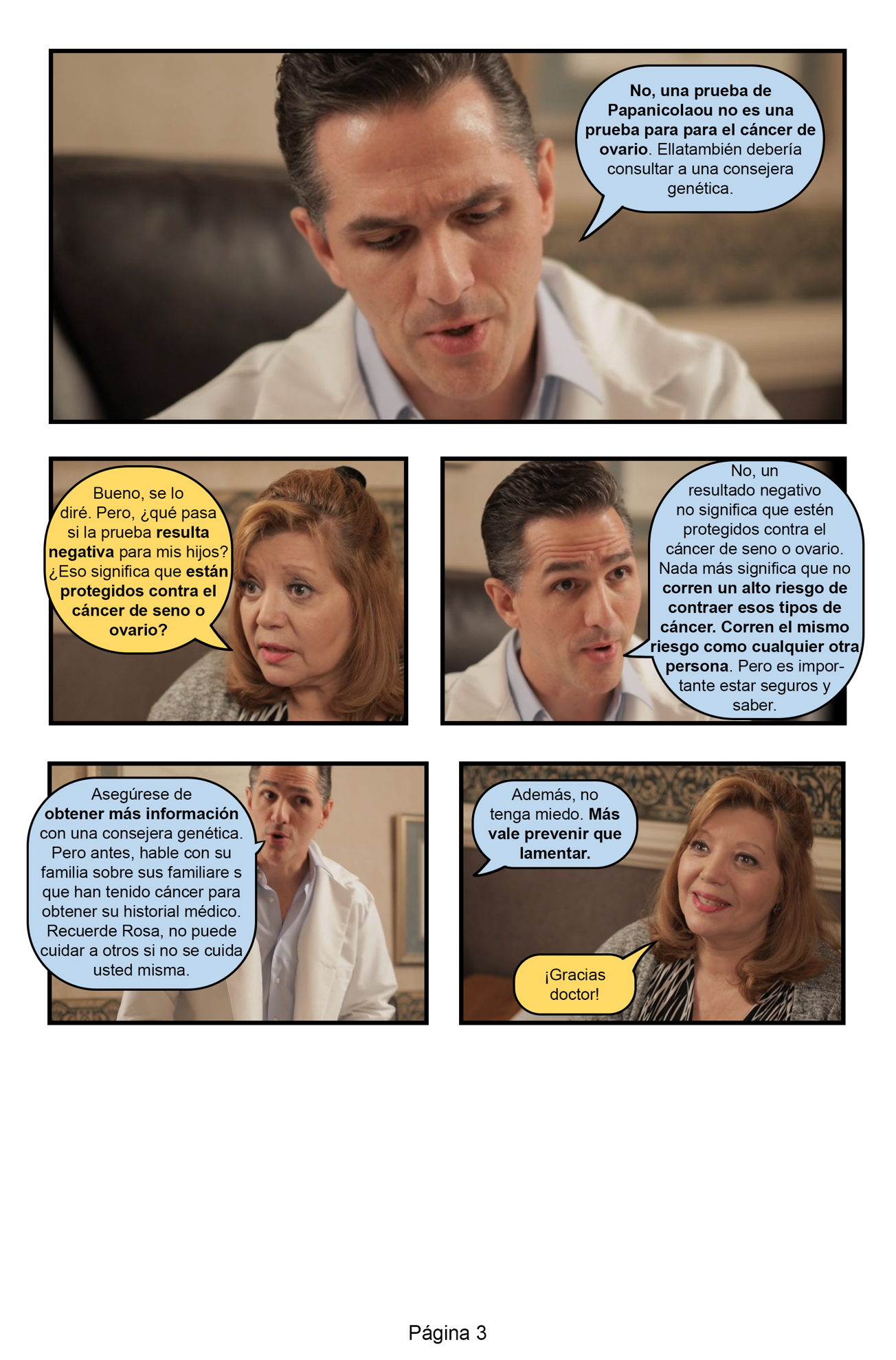
**

1. **Doctor fotonovela – English version**

**
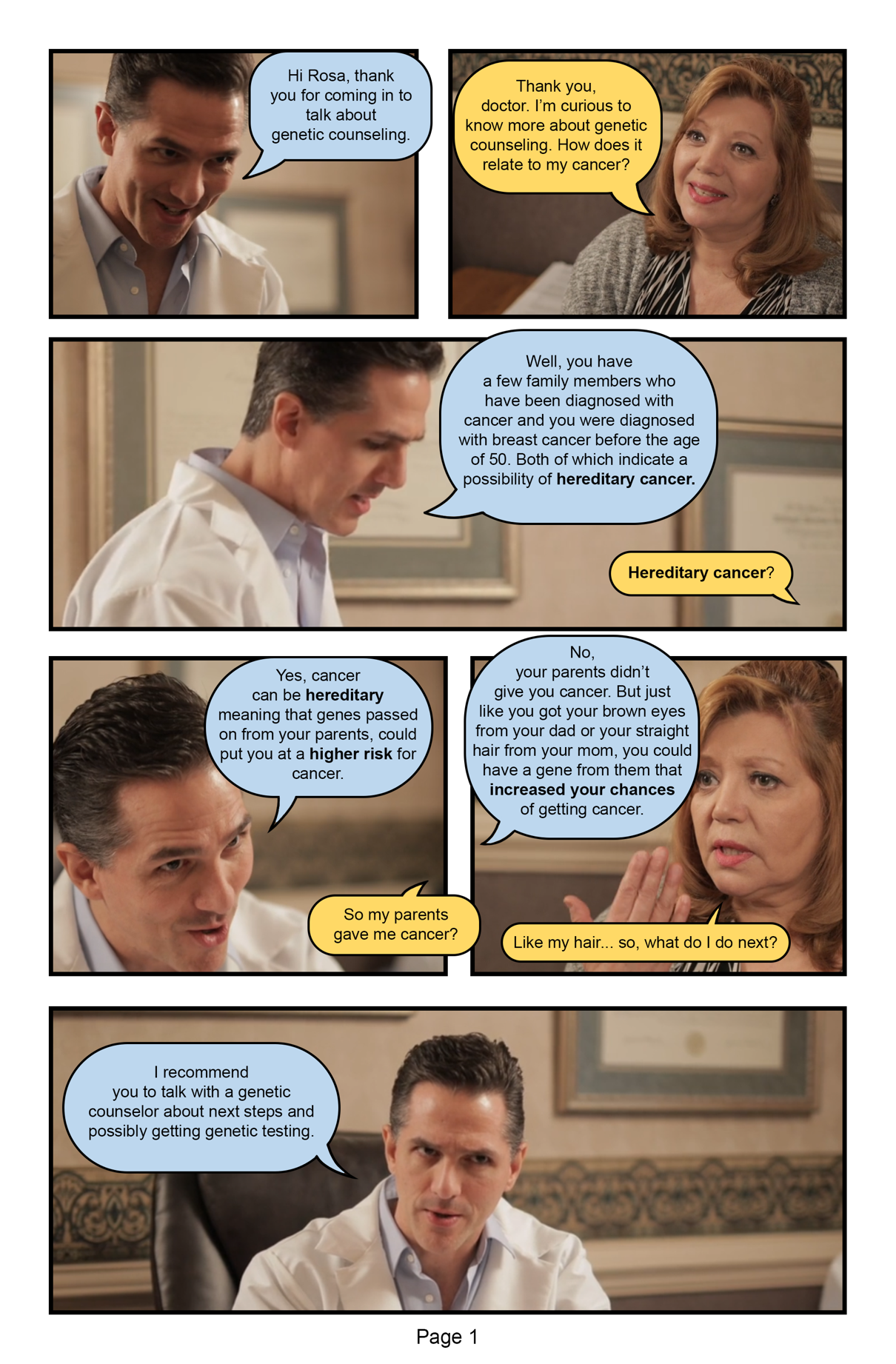
**

**
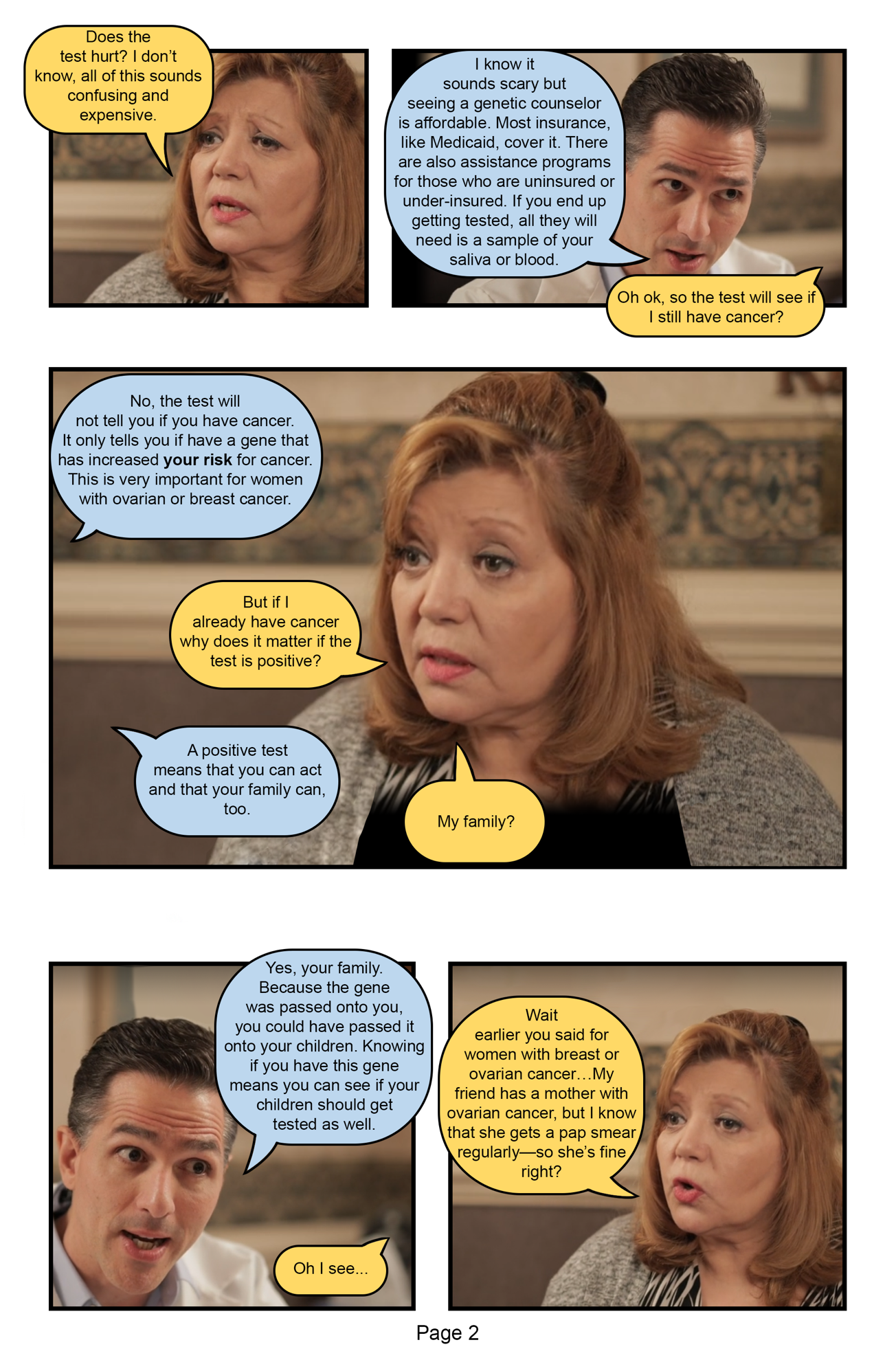
**

**
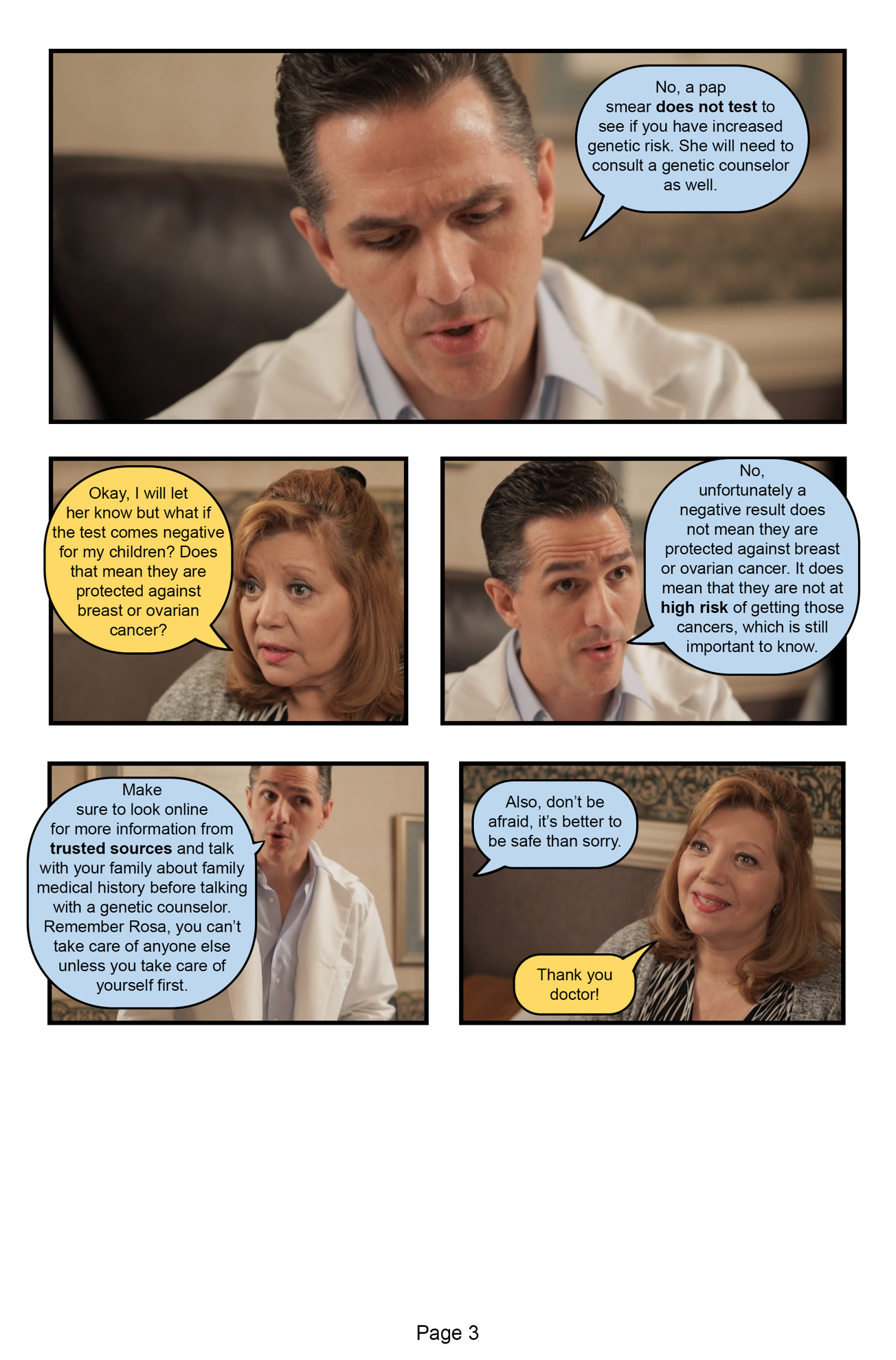
**

1. **Family fotonovela – Spanish version**

**
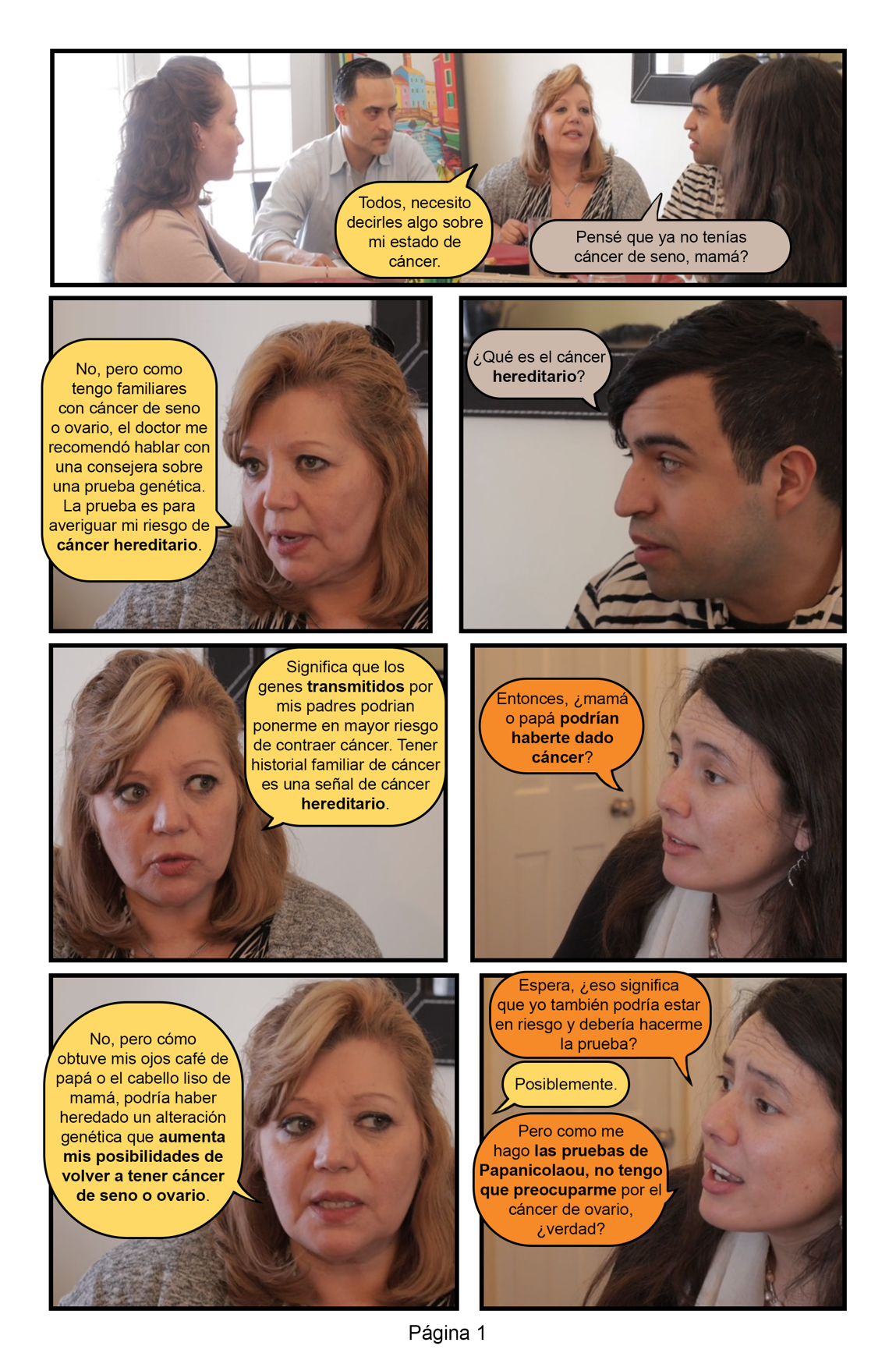
**

**
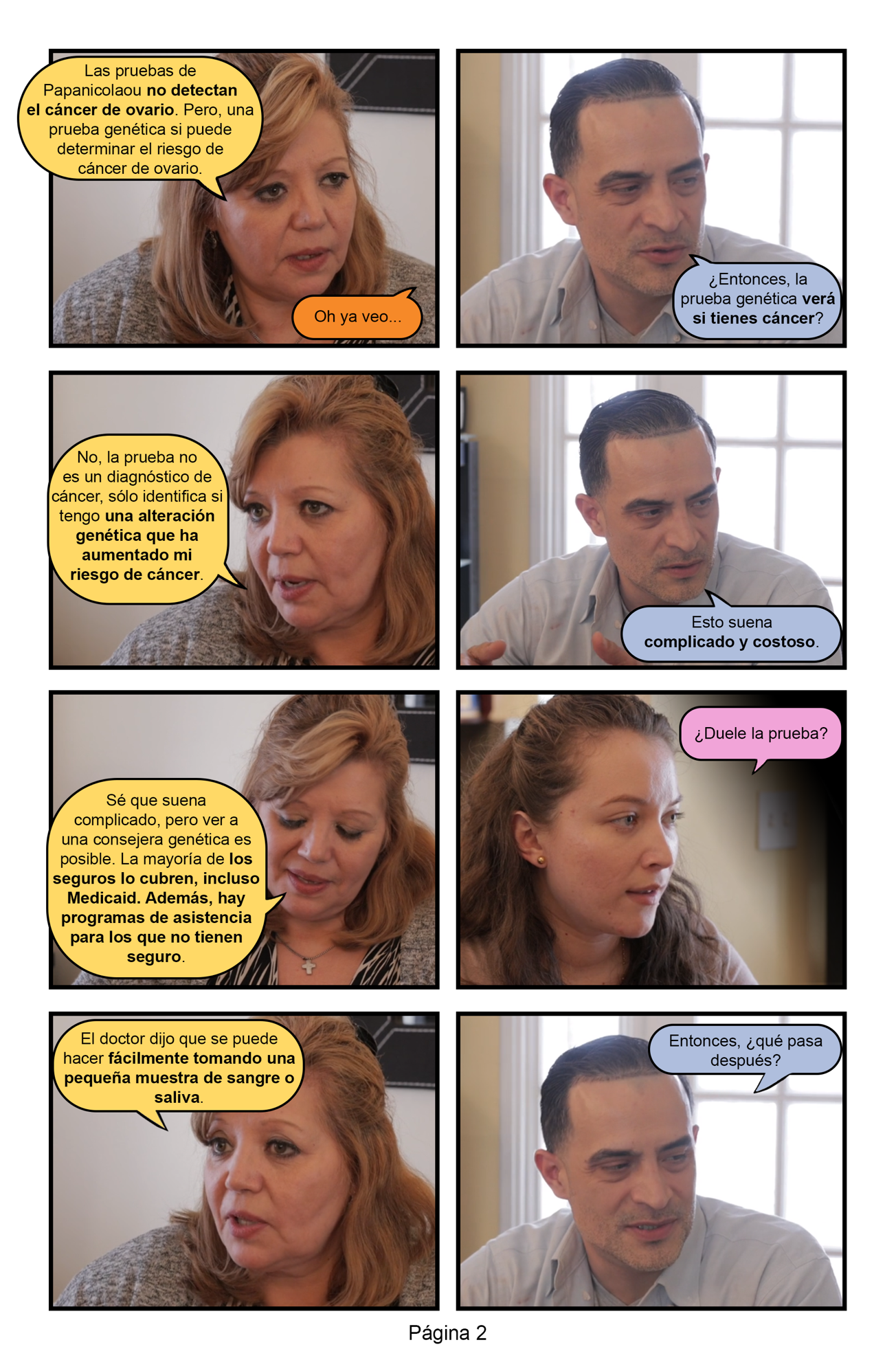
**

**
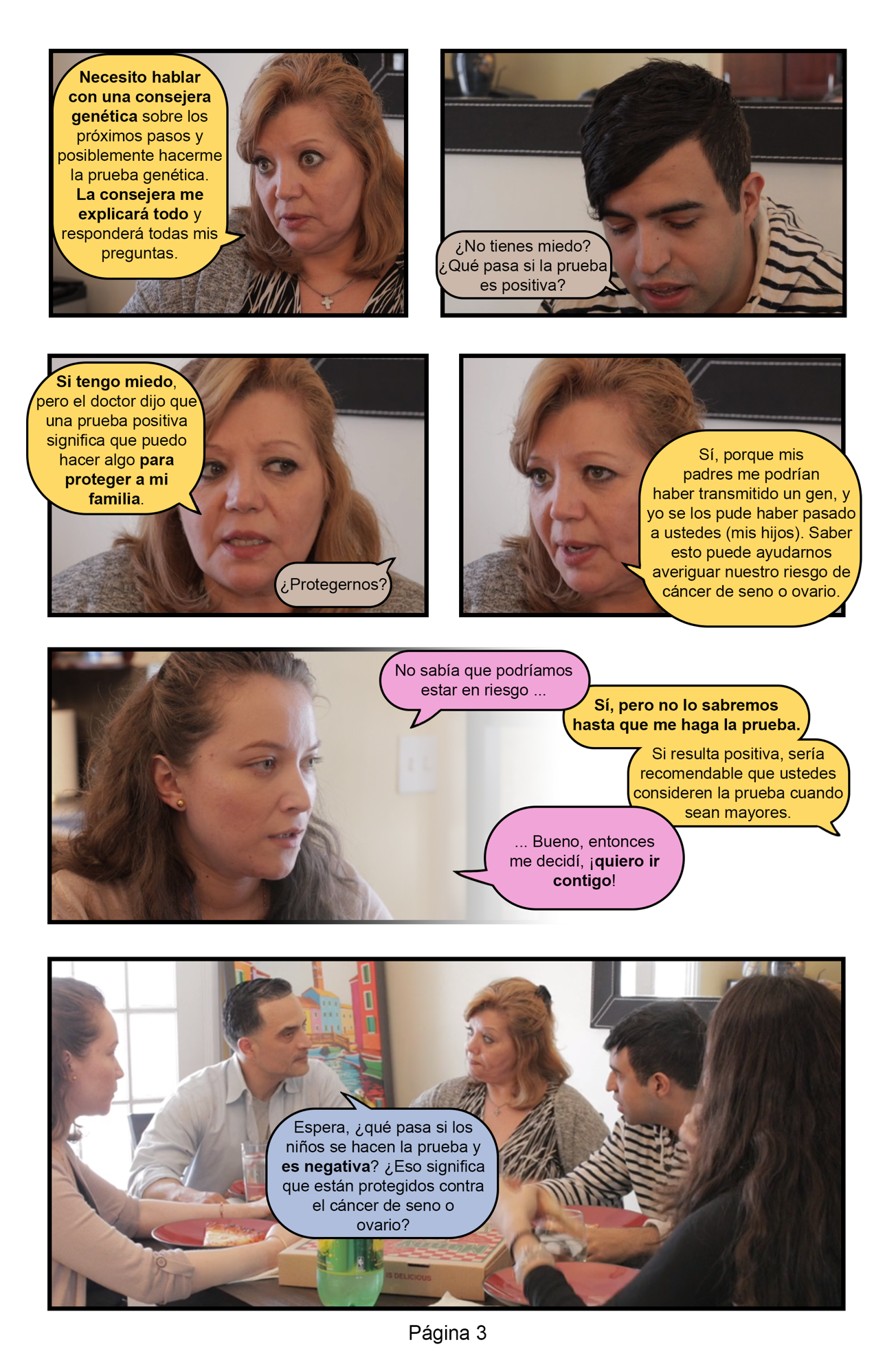
**

**
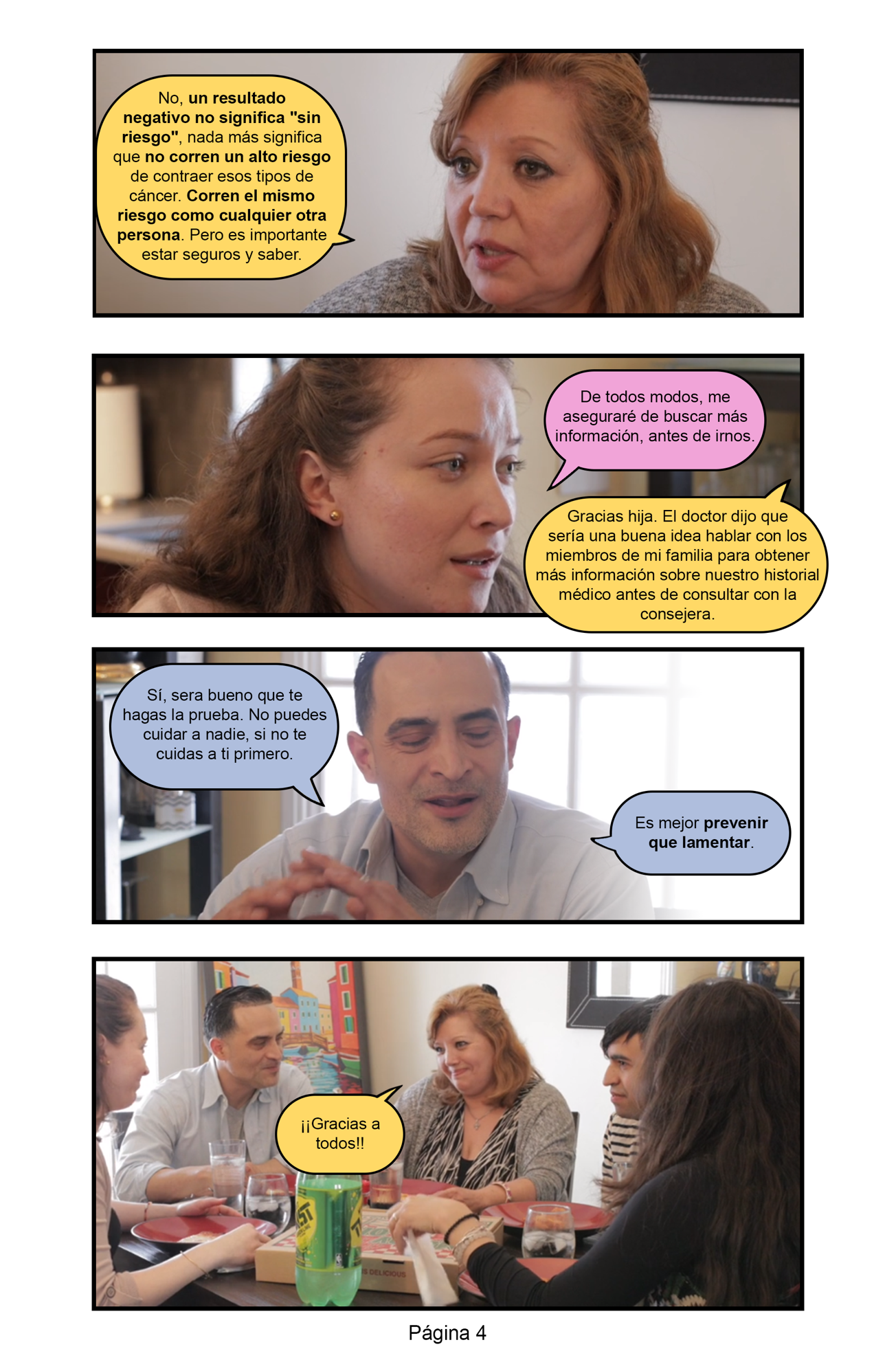
**

1. **Family fotonovela – English version**

**
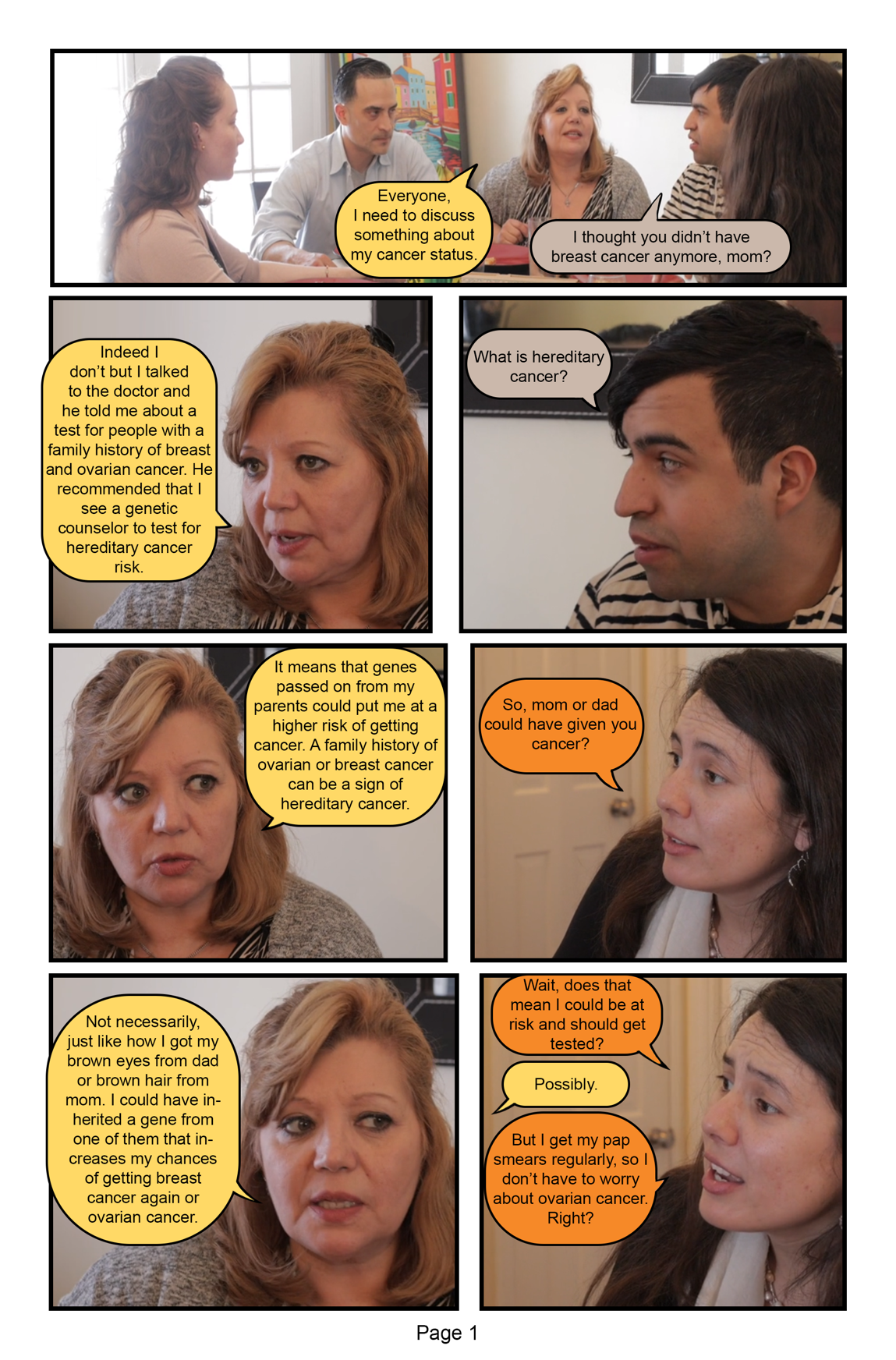
**

**
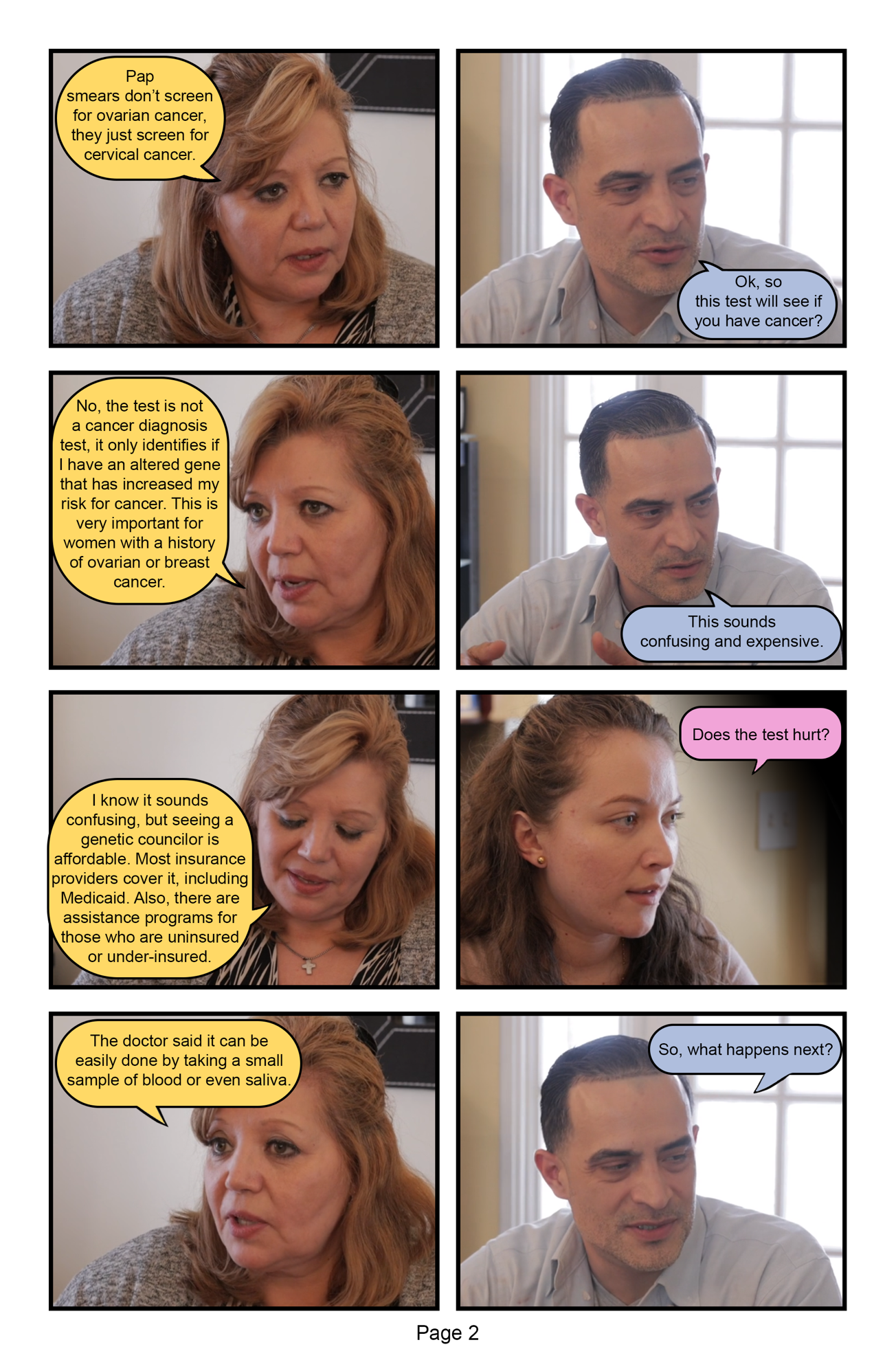
**

**
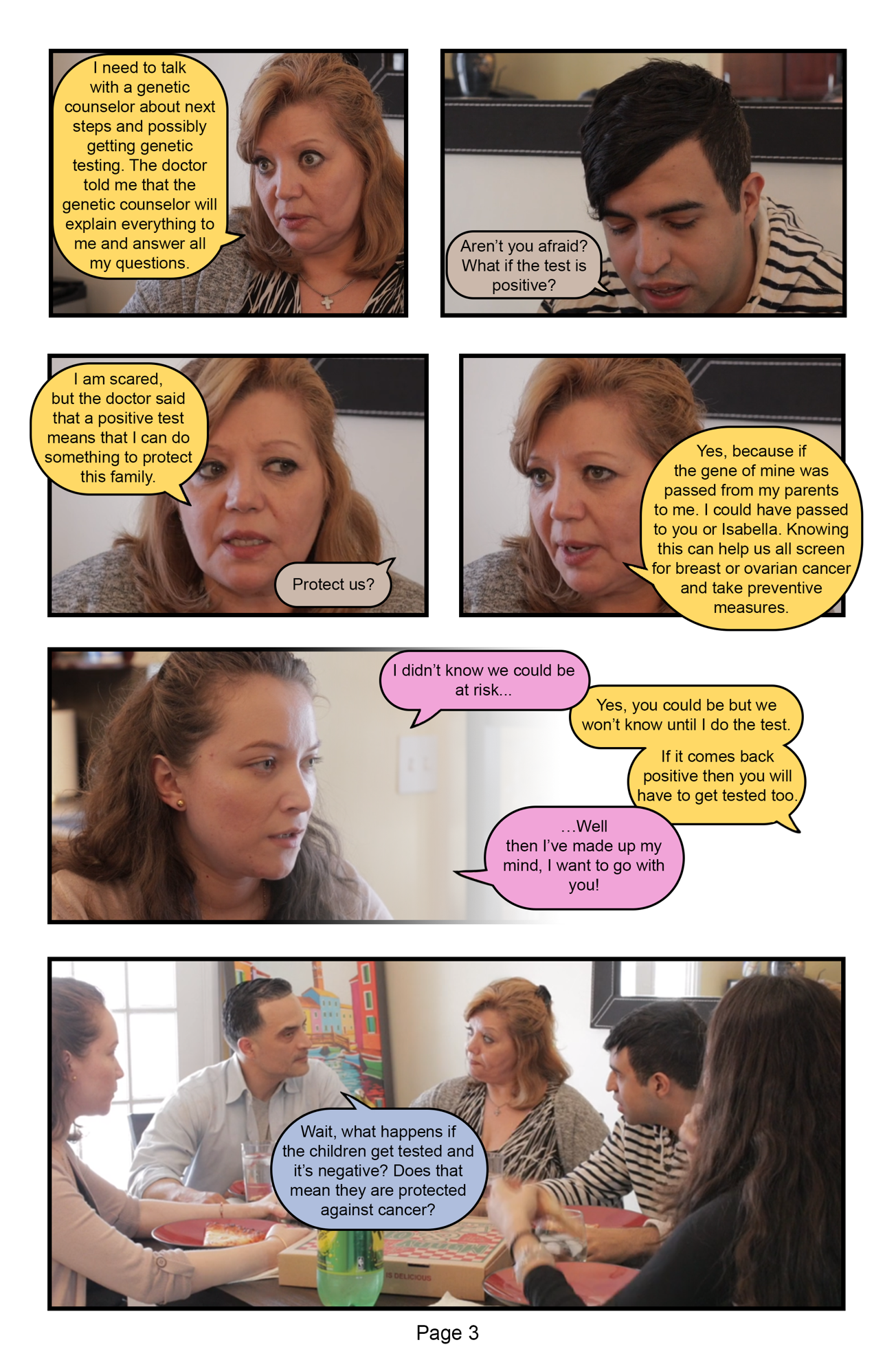
**

**
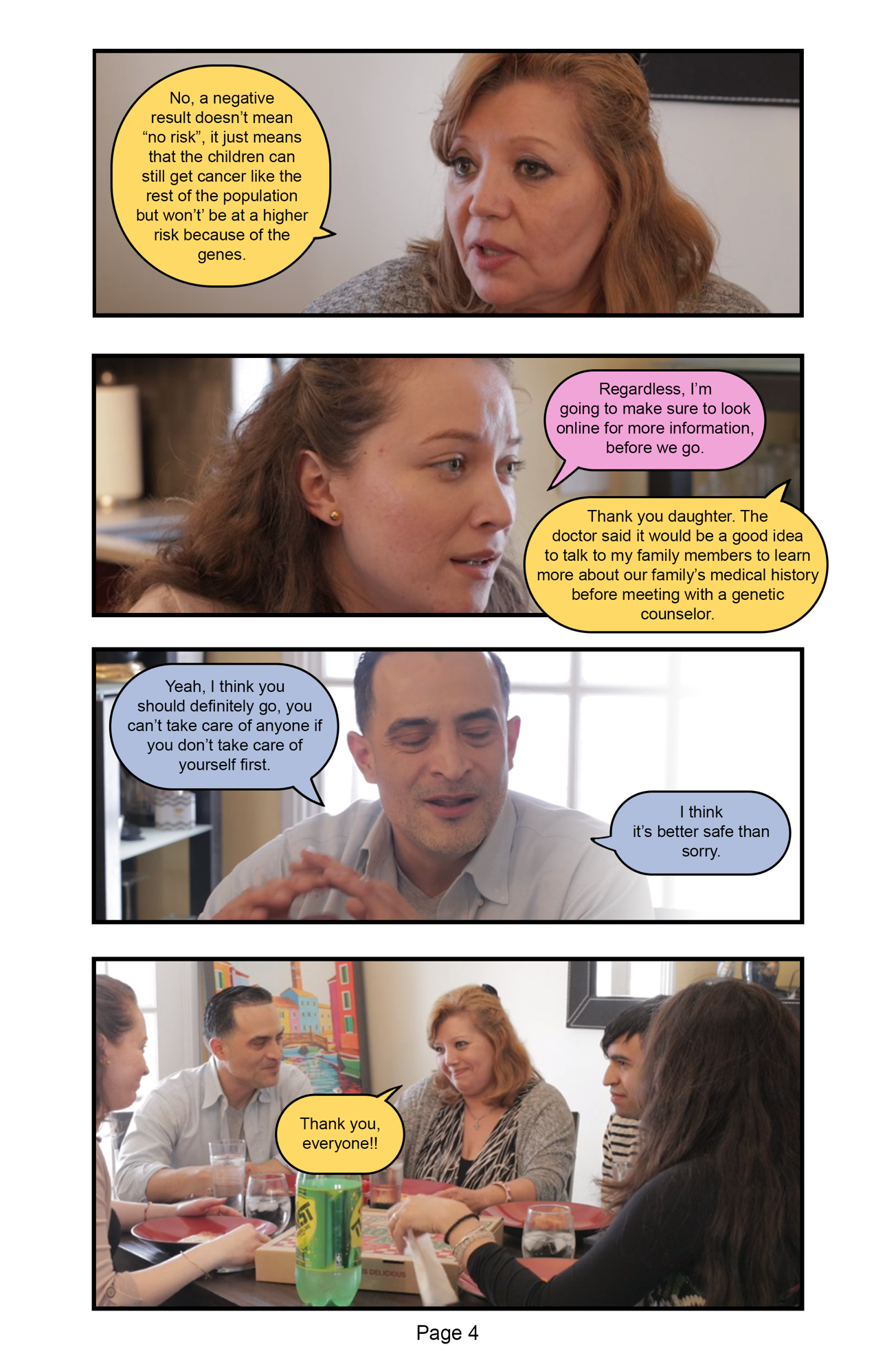
**

**APPENDIX 3**

1. **Friend fotonovela – Spanish version**

**
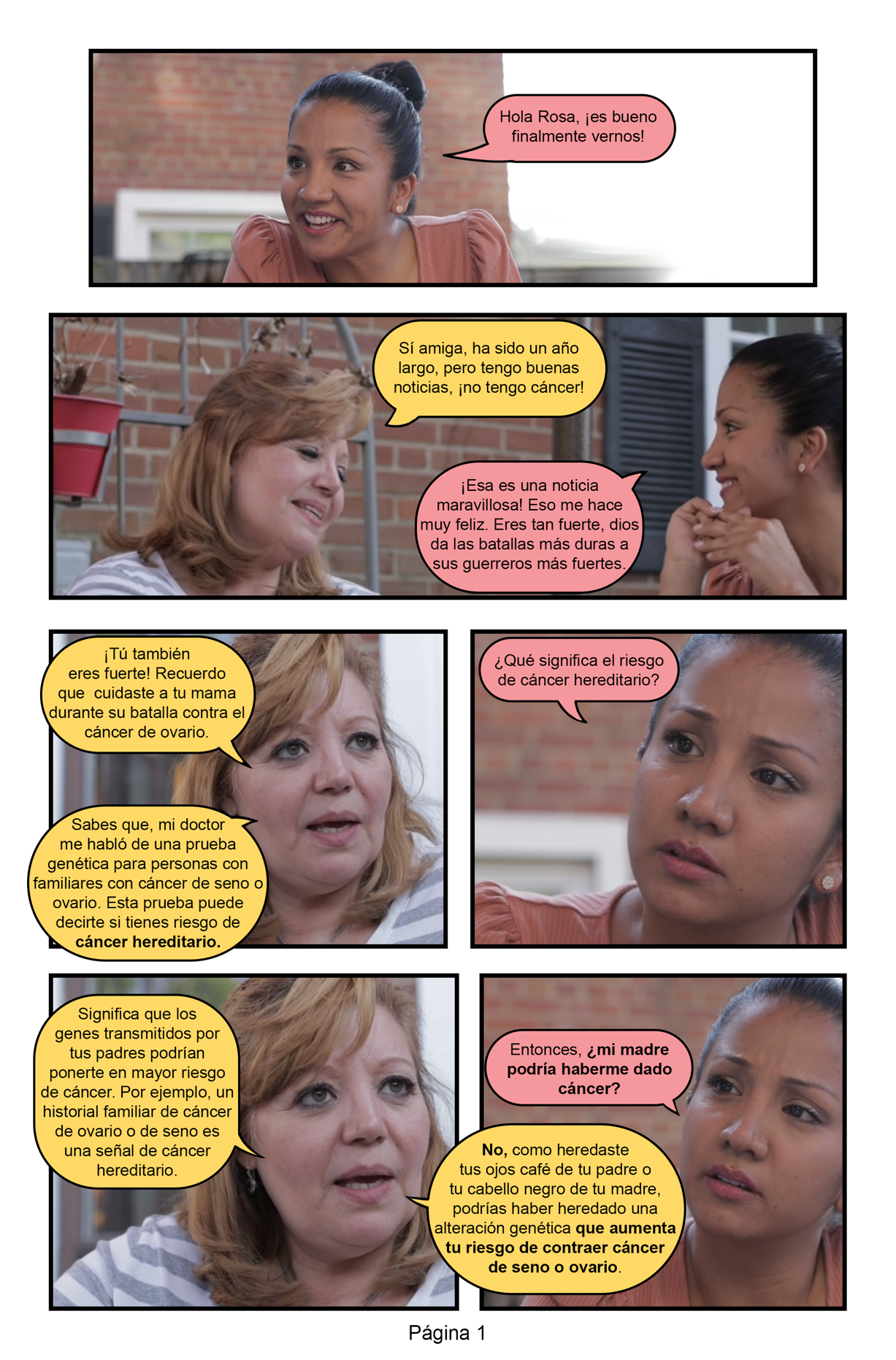
**


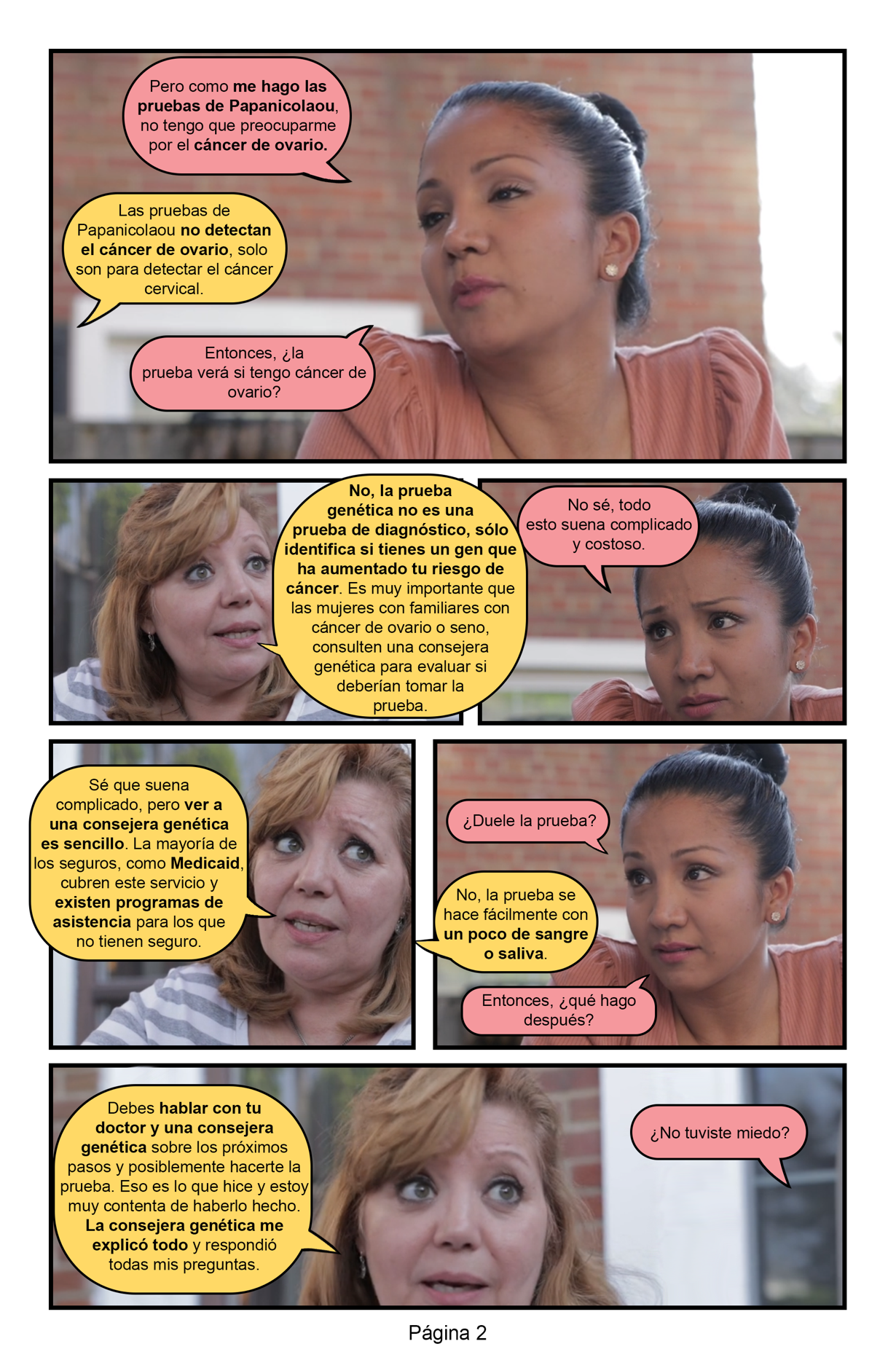


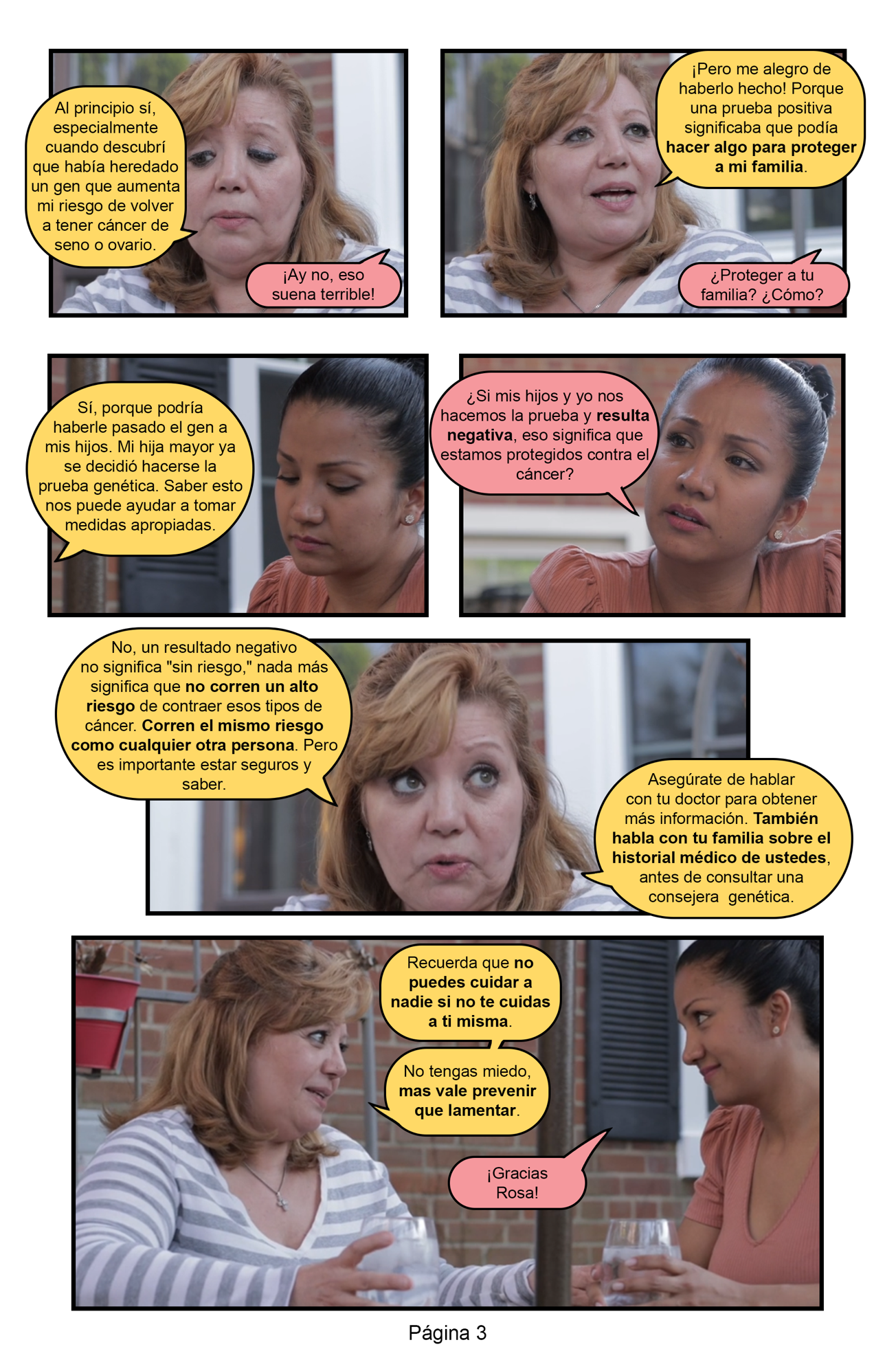


1. **Friend fotonovela – English version**


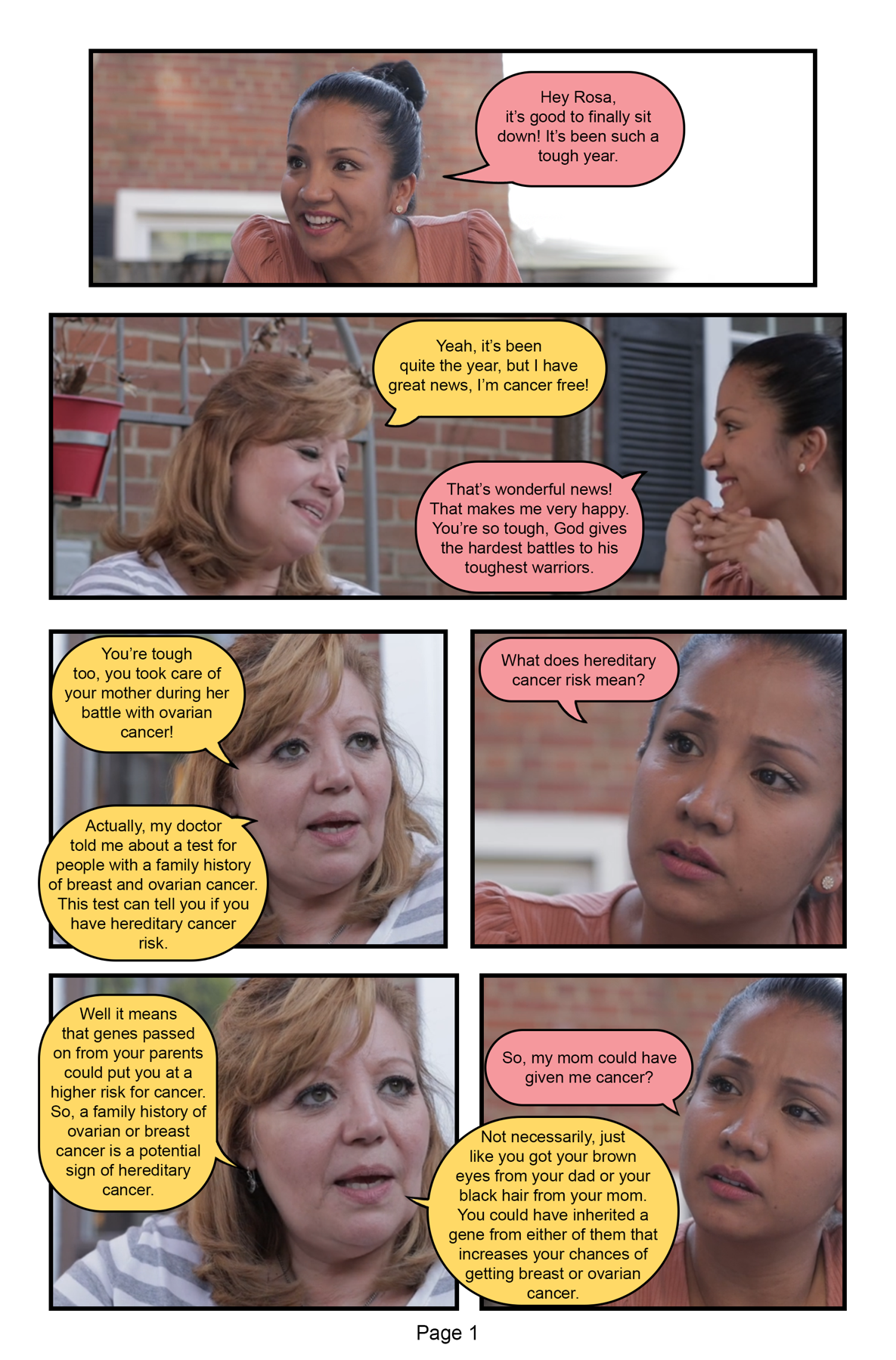


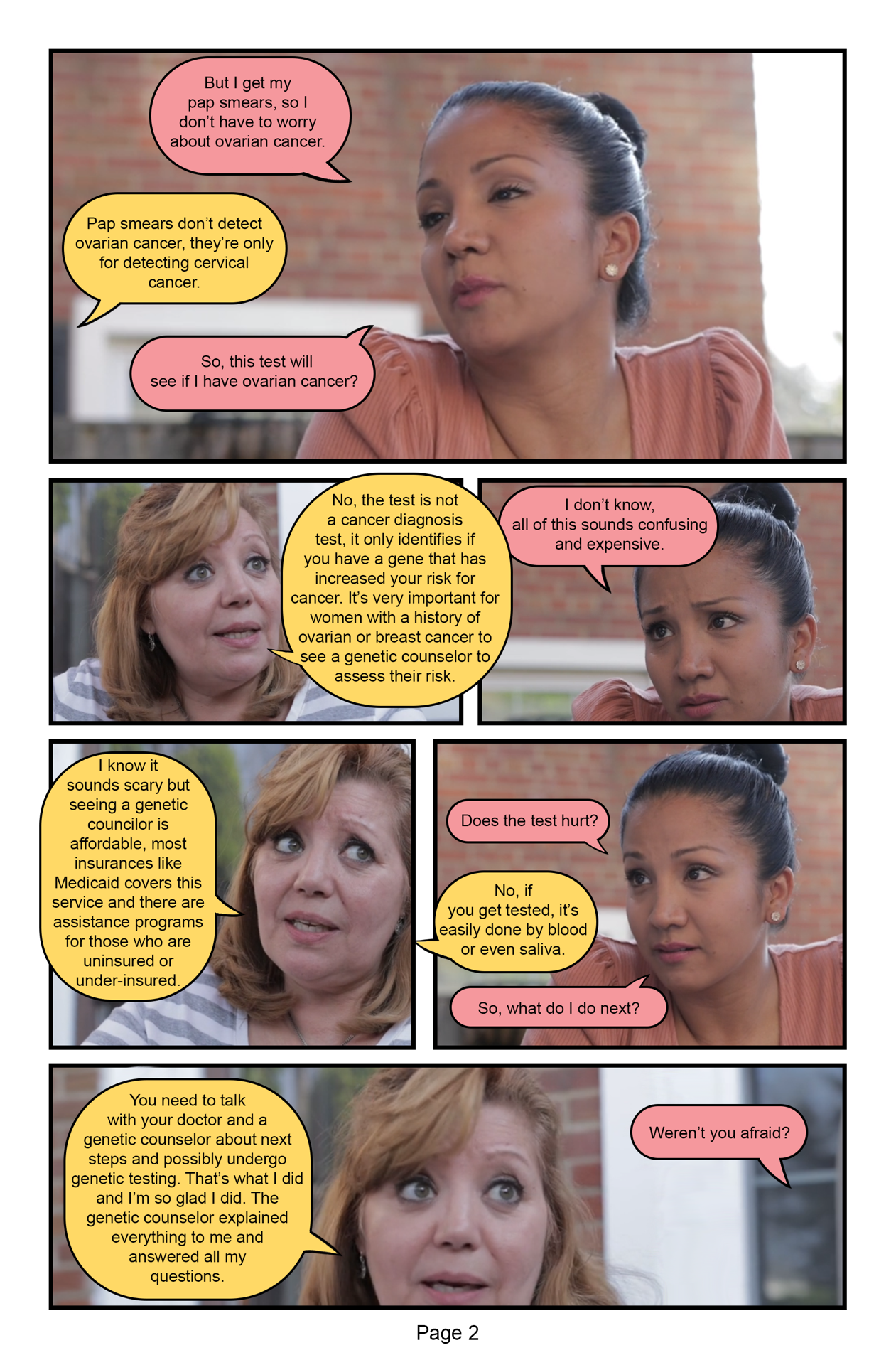

Supplement: Supplementary file 2 — Supplementary Material 2 [file 12687_2024_728_MOESM2_ESM.docx]
