# Supplementary material for: Raising awareness and education of genetic testing and counseling through fotonovelas among Latina women at risk for hereditary breast and ovarian cancer
Source: J Community Genet. 2024 Sep 6;15(5):475–88. doi: 10.1007/s12687-024-00728-5 (PMC11549277; doi:10.1007/s12687-024-00728-5)
Supplement: Supplementary file 3 — Supplementary Material 3 [file 12687_2024_728_MOESM3_ESM.docx]

**Raising Awareness and Education of Genetic Testing and Counseling through Fotonovelas among Latina women at risk for Hereditary Breast or Ovarian Cancer**

**Journal of Community Genetics**

**Author information**

Rolando Barajas, MPH^1,6^, Clara B. Barajas, MPH^2^, Yaideliz M. Romero Ramos^3^, Sara Gómez Trillos, MS^2,4^, Sabrina Sawhney^2^, Claudia Campos^5^, Alejandra Hurtado-de-Mendoza, PhD^2,4^, Melissa Rotunno, PhD^6^, Elizabeth Gillanders, PhD^6^

1. Georgetown University School of Medicine, Washington, DC, USA
2. Georgetown University, Cancer Prevention and Control, Lombardi Comprehensive Cancer Center, Washington, DC, USA
3. Brown University School of Public Health, Department of Epidemiology, Providence, RI, USA
4. Fischer Center for Hereditary Cancers, Washington, DC, USA
5. Nueva Vida, Inc., Alexandria, VA, USA
6. National Health Institutes/National Cancer Institute, Division of Cancer Control and Population Sciences, Genomic Epidemiology Branch, Bethesda, MD, USA

**Corresponding Author:** Clara B. Barajas, [clara.barajas@georgetown.edu](mailto:clara.barajas@georgetown.edu)

**Funding:** This study was funded by the National Health Institutes/National Cancer Institute, Division of Cancer Control and Population Sciences CRAFT Grant.

**APPENDIX 3 – QUESTIONS**

**QUANTITATIVE SCALES**

**LANGUAGE**

1. Which of the following statements best describes how you communicate at home?
   1. I always speak Spanish
   2. I speak more Spanish than English
   3. I speak Spanish as much as I speak English
   4. I speak more English than Spanish
   5. I always speak English
   6. Refused to answer

**WILLINGNESS TO DISCUSS CANCER**

1. On a scale of 1 to 5 (1 =very unlikely, 2 =unlikely, 3 =neutral, 4 =likely, 5 =very likely): How likely are you to talk about cancer with your family?
   1. Why?

**PRECIEVED KNOWLEDGE OF GENETIC COUNSELING AND TESTING**

1. Have you ever heard of genetic counseling or genetic testing?
   [If yes, continue]
   [If no, skip to next question]
   1. How would you rate your knowledge of genetic counseling/testing
      (1 =no knowledge, 2 =some knowledge, 3 =very knowledgeable)?

**WILLINGNESS TO SEEK FUTURE INFORMATION ON GENETIC COUNSELING AND TESTING (asked as a post-fotonvela question to cancer patients and relatives)**

1. On a scale of 1 to 5, how likely are you seek more information about genetic testing/ counseling (1 =very unlikely, 2 =unlikely, 3 =neutral, 4 =likely, 5 =very likely)?

**FOTONOVELA FEEDBACK QUESTIONS**

1. Initial reaction
   1. What are your initial reactions and thoughts on the fotonovela?
      1. How did it make you feel?
   2. What, if anything, about the fotonovela works for you?
      1. Explain
   3. What, if anything, about the fotonovela doesn’t work for you?
      1. Explain
2. Main Idea Recall
   1. What was the main idea the fotonovela was trying to convey?
      1. Why do you say that?
   2. What questions do you have about the information presented to you?
      1. [Probe and provide a few examples (imagery, words, etc.) if participant seems confused or unresponsive]
      2. Example [What do you think of the faces? Do they seem appropriate for the scenario? Why or why not?]
   3. Is any part of the information presented, confusing at all?
      1. Please elaborate
3. Tone
   1. What do you think about the way the conversation/dialogue in the fotonovela sounded?
      1. [To elaborate] Do you think the tone is appropriate for this subject matter?
      2. What, if anything, would you change about the tone to make it more effective?
4. Relatability/Believability
   1. Who do you think this fotonovela trying to reach [To elaborate: who do you think (like group of people) the fotonovela is made for?] Why?
   2. Do you think patients would believe the information that the fotonovela is showing? Why/why not?
      1. Would you change or add anything to make it more believable?
5. Level of Engagement/Memorability
   1. Would you remember this fotonovela?
      1. If so, what parts would stick with you?
   2. Do you feel like the fotonovela taught you anything new?
      1. What did it teach you?
   3. Did the fotonovelas make you think about genetic counseling/testing in a new way?
      1. How so?
6. Format/Look and Feel/Presentation of Information
   1. What do you think about the use of the fotonovela to educate Latinos about genetic counseling/testing?
7. Intentions
   1. How might this information affect *patients* after seeing it? [note: question rephrased as “patients” for health workers or as “you” for cancer patients and relatives]
      1. What do you think *patients* would do after seeing this message?
      2. Do you think it will help motivate *patients* and their families to seek out more information on genetic counseling/testing? Tell me more about that.

**FOTONOVELA COMPARATIVE QUESTIONS**

1. Which fotonovela was the most memorable?
   1. Why was it memorable?
2. Which one was most applicable to you?
   1. Why do you say that?
3. Which fotonovela would you share with a friend or family member?
4. Like, if anything, have any of the fotonovelas influenced you to seek more information about genetic counseling/testing? Tell me more about that.
5. Which one made you more interested in learning more about genetic counseling/testing?
6. Please rank the 3 fotonovelas from your favorite to your least favorite.
7. If you could take one piece from each fotonovela and place it in your favorite fotonovela, what parts would they be?
   1. Can you tell me more about it?
